# Identification of a neuronal transcription factor network involved in medulloblastoma development

**DOI:** 10.1186/2051-5960-1-35

**Published:** 2013-07-11

**Authors:** Maria Łastowska, Hani Al-Afghani, Haya H Al-Balool, Harsh Sheth, Emma Mercer, Jonathan M Coxhead, Chris PF Redfern, Heiko Peters, Alastair D Burt, Mauro Santibanez-Koref, Chris M Bacon, Louis Chesler, Alistair G Rust, David J Adams, Daniel Williamson, Steven C Clifford, Michael S Jackson

**Affiliations:** 1 Institute of Genetic Medicine, Newcastle University, Central Parkway, Newcastle upon Tyne NE1 3BZ, UK; 2 NewGene Limited, Bioscience Building, International Centre for Life, Newcastle upon Tyne NE1 4EP, UK; 3 Northern Institute for Cancer Research, Newcastle University, Newcastle upon Tyne NE1 4LP, UK; 4 Faculty of Medical Sciences, William Leech Building, Newcastle University, Newcastle upon Tyne NE2 4HH, UK; 5 Division of Clinical Studies and Cancer Therapeutics, The Institute of Cancer Research & The Royal Marsden NHS Trust, Sutton, Surrey, SM2 5NG, UK; 6 Experimental Cancer Genetics, Wellcome Trust Sanger Institute, Hinxton CB10 1HH, UK; 7 Department of Pathology, Children’s Memorial Health Institute, Av. Dzieci Polskich 20, 04-730, Warsaw, Poland; 8 Centre for Molecular Oncology, Barts Cancer Institute, Barts and The London School of Medicine and Dentistry, Queen Mary University of London, Charterhouse Square, London EC1M 6BQ, UK; 9 School of Medicine, Faculty of Health Sciences, University of Adelaide, Adelaide, South Australia SA 5045, Australia

**Keywords:** Medulloblastoma, Mutagenesis, Transcription network, Differentiation

## Abstract

**Background:**

Medulloblastomas, the most frequent malignant brain tumours affecting children, comprise at least 4 distinct clinicogenetic subgroups. Aberrant sonic hedgehog (SHH) signalling is observed in approximately 25% of tumours and defines one subgroup. Although alterations in SHH pathway genes (e.g. *PTCH1*, *SUFU*) are observed in many of these tumours, high throughput genomic analyses have identified few other recurring mutations. Here, we have mutagenised the *Ptch*^+/-^ murine tumour model using the Sleeping Beauty transposon system to identify additional genes and pathways involved in SHH subgroup medulloblastoma development.

**Results:**

Mutagenesis significantly increased medulloblastoma frequency and identified 17 candidate cancer genes, including orthologs of genes somatically mutated (*PTEN, CREBBP*) or associated with poor outcome (*PTEN, MYT1L*) in the human disease. Strikingly, these candidate genes were enriched for transcription factors (p*=*2x10^-5^), the majority of which (6/7; *Crebbp*, *Myt1L*, *Nfia, Nfib, Tead1* and *Tgif2*) were linked within a single regulatory network enriched for genes associated with a differentiated neuronal phenotype. Furthermore, activity of this network varied significantly between the human subgroups, was associated with metastatic disease, and predicted poor survival specifically within the SHH subgroup of tumours. *Igf2*, previously implicated in medulloblastoma, was the most differentially expressed gene in murine tumours with network perturbation, and network activity in both mouse and human tumours was characterised by enrichment for multiple gene-sets indicating increased cell proliferation, IGF signalling, MYC target upregulation, and decreased neuronal differentiation.

**Conclusions:**

Collectively, our data support a model of medulloblastoma development in SB-mutagenised *Ptch*^+/-^ mice which involves disruption of a novel transcription factor network leading to *Igf2* upregulation, proliferation of GNPs, and tumour formation. Moreover, our results identify rational therapeutic targets for SHH subgroup tumours, alongside prognostic biomarkers for the identification of poor-risk SHH patients.

## Background

Medulloblastoma (MB) is the most common malignant brain tumour of childhood, accounting for around 10% of paediatric cancer deaths. Despite recent therapeutic advances, up to 40% of patients still die from their disease and cure is often associated with disabling therapy-related effects in later life. New therapeutic approaches based on improved biological understanding of the disease will be essential to improve outcomes, through strategies such as the delivery of risk-adapted therapies guided by molecular prognostic biomarkers, and the stratified use of molecularly-targeted agents (reviewed in [1]).

Four major molecular subtypes of medulloblastoma with distinct biological, clinical and pathological features are now recognised, and this subgrouping is beginning to have clinical impact [1-3]. WNT subgroup tumours (~15% of total) are characterised by activation of the wnt/wingless pathway through *CTNNB1* mutations, and appear to originate from progenitor cells derived from the dorsal brain stem [4]. Patients with WNT-associated tumours have a favourable prognosis and will receive reduced therapies in forthcoming international clinical trials [1,5,6]. The SHH subgroup (~25% of total) is defined by activation of the sonic hedgehog signalling pathway, and mutations in SHH pathway genes (e.g. *PTCH1, SUFU*) arise in a significant subset [2,3]. All evidence suggests that these tumours originate from cerebellar granule neuron precursors (cGNPs) within the external granular layer of the developing cerebellum (reviewed in [7]) or cochlear nuclei of the brainstem [8]. SHH tumours are associated with an intermediate prognosis. Early clinical trials of SHH pathway inhibitors are underway, although acquired resistance has been reported [9] and tumours with downstream pathway mutations (e.g. *GLI2, SUFU*) are predicted to be insensitive to their action [10]. Group 3 and 4 tumours are more heterogeneous and show overlapping molecular features such as frequent chromosome 17 defects. However, Group 3 tumours (~25% of total) have been associated with high-risk features, such as *MYC* gene amplification and large-cell/anaplastic (LCA) pathology, and a poor prognosis [2].

Recent genome-wide studies have further underlined the complexity of MB; heterogeneous mutations targeting processes including histone methylation and chromatin remodelling have been discovered, but these typically describe limited subsets of tumours and few additional recurrent mutations targeting specific genes and/or pathways have been identified [11-15]. Critical genes therefore remain to be uncovered in all MB subgroups and there is a growing need to identify low-frequency alterations which drive disease progression, distinguish these from passenger mutations, and determine their mechanisms of action and clinical significance. Primary tumour data alone may not be sufficient to achieve these goals.

A number of murine MB models have been created allowing comparative analyses (reviewed in [16]), including models which recapitulate WNT [4] and Group 3 [17,18] tumours. However, the most widely studied mouse model is a knockout of the *Shh* transmembrane receptor, *Ptch*[19], which mimics SHH subgroup tumours. *Ptch*^+/-^ heterozygotes develop MBs at a frequency which is significantly influenced by genetic background [20]. Sleeping Beauty *(*SB) murine mutagenesis [21,22], coupled to statistical analysis of insertion site distribution [23,24], has emerged as a powerful method to identify genes involved in a wide variety of human cancers [24-29]. Recently the SB11 transposase [22] driven by the *Math1* promoter has been used to mutagenise developing neuronal tissues in both the *Ptch*^+/-^ and *p53* loss-of-function models of MB [30]. This identified a large number of candidate genes potentially involved in MB progression, and demonstrated that the genetic events observed in metastases show limited overlap with those in matched primary tumours, supporting a bicompartmental genetic model of metastatic disease [30,31].

Here, we report the application of whole-body SB mutagenesis [26] to the *Ptch*^*+/-*^ tumour model, and the identification of 17 genes associated with enhanced medulloblastoma formation. We show that these genes are enriched for neuronal transcription factors defining a novel gene network which, when mutagenised by SB, is associated with increased cell proliferation and reduced neuronal differentiation. Significantly, increased expression of *Igf2*, a gene known to be essential for tumour formation in the *Ptch*^*+/-*^ model, is associated with insertional mutations within this network. Moreover, we show that in human disease, network activity predicts poor survival specifically within the SHH tumour subgroup. Together, these findings provide important novel insights into the molecular mechanisms of medulloblastoma pathogenesis, and identify exploitable therapeutic targets and prognostic biomarkers for development towards improved therapy.

## Results

### The incidence of MB but not RMS is enhanced in mutagenised *Ptch+/-* mice

**Figure 1:**
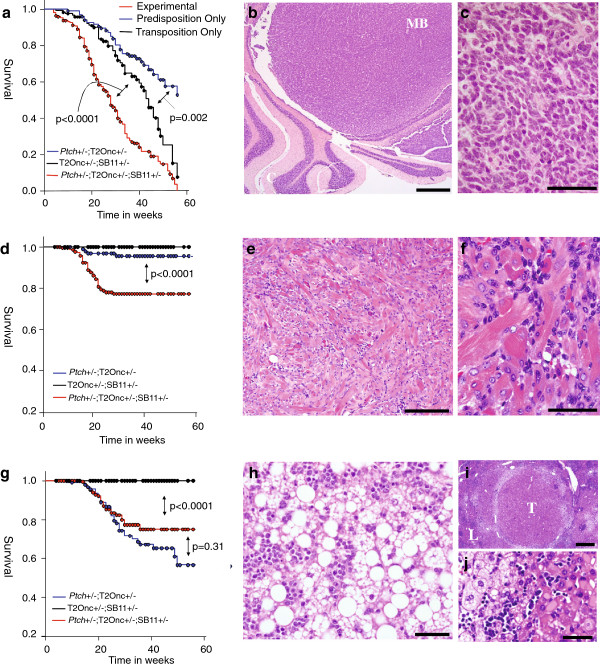
**Mortality in experimental/control cohorts and tumour pathology. a-c.** Kaplan Meier survival curves. All p-values are from log-rank tests. **a.** Mortality due to all causes in experimental cohort and controls. **b.** Medulloblastoma (MB) arising from cerebellum (C). Scale bar = 500 μm. **c.** High resolution image showing typical cytomorphology of small, polygonal or slightly elongated cells. Perivascular pseudorosetting and occasional necrotic foci were also seen in some tumours. Scale bar = 50 μm. **d.** Mortality due to medulloblastoma alone. The difference between predisposition and transposition controls was not significant (p=0.118). **e.** Typical appearance of RMS consisting of variable numbers of small, round mitotically active cells together with large more well-differentiated rhabdomyoblasts arranged in interlacing fascicles. Scale bar = 200 μm. **f.** High resolution image showing large rhabdomyoblasts with characteristic cross-striations, accompanied by smaller less well differentiated cells. Scale bar = 50 μm. **g.** Mortality due to RMS alone. **h.** Hepatocellular adenoma containing mixed pattern (micro- and macrovesicular) steatosis and leukaemic infiltration. Scale bar = 50 μm. **i.** Low power view of a well demarcated hepatocellular carcinoma with leukaemic infiltration (T=tumour, L=liver parenchyma). Scale bar = 500 μm. **j.** High resolution image of hepatocellular carcinoma showing tumour cells (right-hand side of image) with eosinophilic cytoplasmic inclusions. Scale bar = 50 μm. All sections are stained with Haematoxylin and Eosin.

A total of 243 mutagenised *Ptch*+/- animals (*Ptch*+/-; SB11+/-;T2Onc+/-) and 195 control littermates were aged for up to 15 months and monitored for tumour development. Mortality in mutagenised *Ptch*+/- animals was approximately 90% after 1 year, significantly higher than in the predisposition only (*Ptch+/-*;T2Onc+/-) or transposition only (T2Onc+/-;SB11+/-) control genotypes (Figure 1a and Additional file 1: Table S1). Approximately ~28% of mutagenised *Ptch*+/- animals aged for more than 6 months succumbed to haematological neoplasms, which usually presented as large thoracic tumours and/or hepatosplenomegaly. A low frequency of parenchymal brain lesions consistent with glial tumours (2%) was also observed. These malignancies occurred at comparable frequencies within the transposition controls, have previously been reported using the same transposase/transposon combination [26,29], and are not analysed further here.

Despite this mutagen-specific burden, significant mortality within mutagenised *Ptch*+/- animals was related to the *Ptch* genotype. Large exophytic and/or invasive MBs (Figure 1b and c) developed in ~23% of mutagenised *Ptch*+/- animals aged for over 6 months, with no macroscopic meningeal masses being observed (typical of the *Ptch* model [19]). Tumours with indistinguishable pathology were observed in the predisposition controls but at a much lower frequency (6%), with survival analysis providing clear evidence that SB mutagenesis enhanced the predisposition of *Ptch*+/- mice to MB (p<0.0001, Figure 1d). Slow growing and locally invasive rhabdomyosarcomas (RMSs), also typical of the *Ptch*+/- model [32], developed in ~22% of experimental animals aged over 6 months (Figure 1e and f). However, mutagenesis did not significantly alter RMS related mortality relative to predisposition controls (Figure 1g, p=0.31). Finally, multiple liver tumours (Figure 1h-j), morphologically similar to those generated using a conditional SB11 screen for hepatocellular carcinoma [27], were observed in 19 mutagenised *Ptch*+/- mice but not in predisposition controls. Although not previously reported in whole body SB mutagenesis screens, liver tumours were also observed at low frequency in transposition only controls (Additional file 1: Table S1), suggesting that they can be induced by SB mutagenesis alone.

### SB insertions target neuronal transcription factors

To identify genes responsible for the impact of mutagenesis upon MB incidence, transposon insertion sites within MBs and control cerebellar tissues were recovered using Splinkerette PCR, sequenced, and their distribution analysed using Gaussian kernel convolution (GKC [23]) to identify common insertion sites (CISs, see Methods). A total of 17 genes were identified within 20 CISs recovered [median p-value = 0.008 (Table 1)]. The majority were also recovered using Monte Carlo simulation analysis [24], and these are highlighted in bold in Table 1. Three of the CISs target known tumourigenic genes (*Crebbp*, *Nfib* and *Pten*), mutations within orthologs of two (*Crebbp* and *Pten*) are somatically mutated at low frequency in human MB [11,33], and six (*Crebbp*, *Nfia*, *Nfib*, *Pten*, *Sfi1,* and *Tead1*) have recently been identified as MB CISs in a tissue-specific mutagenesis screen [30]. Strikingly, ontological analysis established that six of the 17 CIS genes have transcription factor activity (*Nfia*, *Tead1*, *Tgif2*, *Nfib*, *Myt1l* and *L3mbtl4*), a highly significant excess relative to expectation (FDR corrected p-value = 2×10^-5^). Furthermore, seven genes (*Tgif2*, *Pten*, *Nfia*, *Nfib*, *Myt1l*, *Slit3* and *Fgf13*) are implicated in neuronal biological processes. In contrast, only three genic CISs were identified in control tissue, all with modest p-values (0.01-0.05, Table 1). All of these observations are consistent with insertional mutations at CISs contributing to increased penetrance of the tumour phenotype. As all SB insertions within *Tgif2* mapped to a single ~4 kb intron, these were analysed in detail using both genomic DNA and cDNA templates. This both validated our sequence data and confirmed the inferred upregulation of this gene by SB insertion (Additional file 2: Figure S1).

**Table 1:** Common Insertion Sites (CISs) in Medulloblastomas and Cerebellum Controls

**Gene**	**Chr**	**CIS width**	**N**	**I**	**GKC p-value**	**Other genes**	**Inferred MOA**	**CGC**	**MB- Mut**	**MB- CIS**
**A. Medulloblastomas**										
***Nfia***	4	28.6	20	27	<1E-14	-	Loss	-	-	Ptch
***Atxn2***	5	25	6	6	<1E-14	-	?	-	-	-
***Tead1***	7	19.1	6	7	3.9E-14	-	Gain	-	-	Ptch
***Tgif2***	2	0.8	3	4	0.0006	-	Gain	-	-	-
***Crebbp***	16	37.1	5	5	0.0009	-	Loss	S	Y	Ptch
***Dscr3***	16	18.3	5	6	0.0009	-	Loss	-	-	-
**CIS13:72336914**	13	44.3	5	6	0.0042	n/a	n/a	n/a	n/a	n/a
Pten*	19	50.2	4	5	0.0046	-	Loss	S/G	Y	Both
***Itgbl1***	14	43.4	5	5	0.0062	*Fgf14*	?	-	-	-
***Nfib***	4	24.7	7	9	0.0071	-	Gain	S	-	Ptch
*Myt1l*	12	67.2	8	10	0.0089	*Pxdn,Tpo,Sntg2*	Loss	-	-	-
*Ankrd5*	2	1071.3	8	15	0.0092	*Plcb4, Pak7,Snap25*	?	-	-	-
*Slit3*	11	494.3	8	12	0.012	-	Loss	-	-	-
***Tmem45b***	9	58.4	4	5	0.019	-	?	-	-	-
***Sfi1***	11	51	5	6	0.021	-	Loss	-	-	Both
**CIS15:70979306**	15	20.3	4	4	0.028	n/a	n/a	n/a	n/a	n/a
*Fgf13*	X	23.5	3	4	0.026	-	Gain	-	-	-
CIS3:147532546	3	140.5	4	7	0.043	n/a	n/a	n/a	n/a	n/a
*L3mbtl4*	17	221.6	7	7	0.043	*Tmem200c*	Loss	-	-	-
*Adcy5*	16	127.1	4	6	0.044	*Ptblb*	Loss	-	-	-
**B. Cerebellum controls**										
***Faf1***	4	41.7	4	4	0.01	-	?			
***l7Rn6***	7	10	3	3	0.018	*Ccdc81*	?			
CIS6:31346217_240k	6	844	6	8	0.045	n/a	n/a			
*Ric3*	7	99	4	4	0.035	*Tub*	?			
CIS9:68415409_240k	9	1738	9	11	0.016	n/a	n/a			

Genes associated with CISs, the chromosomes to which they map, and the genomic extent of each CIS are shown. GKC p-values are from the Genome Kernel Convolution analysis (see Methods). The number of tumours **(N)** and T2Onc insertions **(I)** which define each GKC CIS, together with other genes within the CIS interval, are also shown. The position and orientation of SB insertions can be used to infer the mode of action (**MOA**) of insertion (e.g. [28]). Most of the CISs have insertions distributed throughout the genes they identify, and in both orientations, suggesting loss of function **(Loss)**. Both of the CIS genes which have proven tumour suppressor activity (*Pten* and *Crebbp*) show this pattern. An excess of inserts in the 5’ end of a gene, and in a +ve orientation with respect to transcription, implies upregulation of expression **(Gain)** through SB enhancer function [34]. **CGC** – Genes known to cause cancer due to somatic (**S**) or germline (**G**) mutations from the Cancer Genome Census (http://www.sanger.ac.uk/genetics/CGP/Census/). **MB-Mut** – Somatic mutations previously identified in MB [11,12,14,15]. **MB-CIS** – previously identified as an MB CIS [30] in either the Ptch model (**Ptch**) or the Ptch and p53 model (**both**). *Pten, was also identified as a CIS within haematological tumours (data not shown) but is included here as it is a known MB gene. For details, see Methods.

### Association of CIS genes with survival and focal copy number alterations

**Figure 2:**
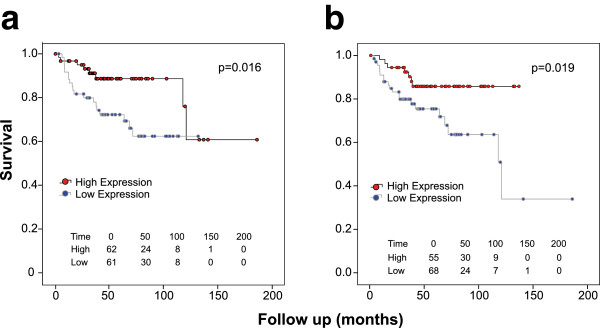
**Expression of *MYT1L *and *PTEN *correlates with survival.** Kaplan Meier curves showing survival in high and low expressing groups in data of [35] split by median of expression in all MB regardless of subtype. **a**. *MYT1L*, **b**. *PTEN*. Numbers at risk and log-rank test p-values are shown.

To investigate the association between CIS gene expression and survival, log-rank (Mantel-Cox) tests were performed on median split microarray expression data from human tumours [35]. Reduced expression of two genes, *PTEN* (a tumour suppressor previously implicated in MB) and *MYT1L*, was associated with poor outcome (Figure 2). This is consistent with the inferred mode of action of the SB insertions that target these genes (Table 1). Furthermore, the association of *MYT1L* expression with survival remained significant within a Cox-regression model incorporating high-risk clinical features using the data from Cho, Tsherniak *et al*[35], even after exclusion of the good prognosis WNT subgroup (p=0.011, see Additional file 3: Table S2).

To investigate the relationship between CIS genes and regions of chromosomal loss or gain defined within primary tumours [35,36], a GISTIC analysis was also performed [37]: *PTEN* maps within a well-established region of common chromosomal loss on 10q associated with SHH tumours [38], and both *NFIB* and *TMEM45B* were found to be present within peak regions of localised copy number gain (Additional file 4: Figure S2). While this is consistent with the mode of action inferred for *PTEN* and *NFIB*, the mode of action of *TMEM45B* remains unclear from insert data alone (Table 1).

### CIS genes are differentially expressed in MB clinico- genetic subgroups

To establish whether the CIS genes are relevant specifically to the SHH subgroup of tumours, we used published data sets [38,39] to compare expression of human orthologs in SHH subgroup tumours with expression in all other subgroups. Of the 17 CIS genes, 9 show significant differential expression when SHH subgroup tumours are compared to all others (including *MYT1L* and *PTEN* discussed above), and 15 show differential expression in one or more clinicogenetic subgroups (Table 2). However, only two (*ITGBL1* and *L3MBTL4*) show clear differential expression in the SHH subgroup alone. Some show marked differential expression in a single non-SHH subgroup, such as *NFIA* (Group 3 tumours) and *FGF13* (Group 4 tumours), or in more than one subgroup (e.g. *TGIF2* and *MYT1L*), suggesting that these genes may be relevant to MB in general. We also investigated expression with respect to the presence/absence of metastatic disease, and 7 genes show a significant association, most notably genes with extreme expression values in Group 3 and 4 tumours where metastatic disease is common (Table 2).

**Table 2:** CIS gene expression according to clinicogenetic groups and presence of metastatic disease

**Clinicogenetic groups**																			**Metastatic disease**						
		**SHH v Other Subgroups**				**WNT (N=15)**			**SHH (N=24)**			**Group 3 (N=24)**			**Group 4 (N=48)**				**Mets (N=32)**			**No Mets (n=75)**			
		**Mean SHH**	**Mean others**	**t**	**p-val**	**Mean**		**SE**	**Mean**		**SE**	**Mean**		**SE**	**Mean**		**SE**	**p-val**	**Mean**		**SE**	**Mean**		**SE**	**p-val**
*NFIA*	22497_at	11.25	10.80	-1.31		11.41	±	0.18	11.25	±	0.30	9.44	±	0.42	11.32	±	0.16	**	10.78	±	0.32	11.03	±	0.15	
*ATXN2*	202622_s_at	7.41	7.60	2.00		7.50	±	0.11	7.41	±	0.08	7.36.	±	0.11	7.75	±	0.06	**	7.68	±	0.08	7.47	±	0.05	*
*TEAD1*	224955_at	10.80	9.85	-6.34	**	11.10	±	0.12	10.80	±	0.12	10.13	±	0.15	9.33	±	0.07	**	9.68	±	0.13	10.11	±	0.11	*
*TGIF2*	216262_s_at	7.61	5.02	-8.98	***	7.75	±	0.10	7.61	±	0.23	5.48	±	0.28	3.97	±	0.10	**	4.46	±	0.22	5.88	±	0.22	***
*CREBBP*	202160_at	9.93	9.80	-0.85		9.95	±	0.10	9.93	±	0.10	9.13	±	0.32	10.09	±	0.07	**	9.76	±	0.13	9.82	±	0.12	
*DSCR3*	203635_at	7.08	6.78	-3.19	**	6.84	±	0.18	7.08	±	0.08	6.69	±	0.09	6.80	±	0.06	**	6.82	±	0.08	6.82	±	0.06	
*PTEN*	225363_at	10.31	11.12	4.63	***	11.16	±	0.20	10.31	±	0.15	10.12	±	0.15	11.62	±	0.06	**	11.03	±	0.14	10.94	±	0.10	
*ITGBL1*	214927_at	6.49	3.90	-4.47	***	4.27	±	0.56	6.49	±	0.55	3.16	±	0.25	4.16	±	0.25	**	3.91	±	0.33	4.85	±	0.27	*
*NFIB*	209290_s_at	12.92	12.67	-0.66		10.46	±	0.48	12.92	±	0.32	12.18	±	0.38	13.58	±	0.09	**	12.81	±	0.28	12.85	±	0.16	
*MYT1L*	210016_at	8.27	10.24	2.80	**	6.06	±	0.70	8.27	±	0.64	9.20	±	0.43	12.02	±	0.11	**	11.28	±	0.22	9.41	±	0.36	**
*ANKRD5*	220144_s_at	4.63	4.75	0.54		4.51	±	0.28	4.63	±	0.19	5.46	±	0.21	4.46	±	0.10	**	4.65	±	0.16	4.77	±	0.11	
*SLIT3*	203813_s_at	3.01	2.98	-0.17		2.90	±	0.09	3.01	±	0.14	2.82	±	0.05	3.09	±	0.10		2.98	±	0.10	2.94	±	0.06	
*TMEM45B*	226226_at	2.37	2.44	1.57		2.34	±	0.04	2.37	±	0.04	2.52	±	0.04	2.44	±	0.03	*	2.45	±	0.03	2.44	±	0.02	
*SFI1*	36545_s_ar	5.96	5.19	-2.68		5.74	±	0.24	5.96	±	0.25	4.42	±	0.23	5.42	±	0.19	**	5.24	±	0.20	5.36	±	0.16	
*FGF13*	205110_s_at	5.88	8.10	6.66	*	5.33	±	0.86	5.88	±	0.54	6.81	±	0.49	9.58	±	0.16	**	8.73	±	0.39	7.01	±	0.32	**
*L3MBTL4*	228557_at	6.85	3.73	-7.43	***	4.28	±	0.28	6.85	±	0.40	4.82	±	0.22	3.01	±	0.10	**	3.76	±	0.21	4.59	±	0.24	*
*ADCY5*	228182_at	3.50	3.70	0.66	***	3.52	±	0.27	3.50	±	0.27	2.73	±	0.16	4.25	±	0.21	**	3.65	±	0.22	3.60	±	0.16	

Genes and associated Affymetrix probe IDs are shown. All values are mean log2 expression levels from [38] and [39]. Standard errors (SE) are shown. SHH v Other Subgroups, and presence/absence of metastatic disease, were analysed using t-tests. Clinicogenetic groups [2] were analysed using ANOVA. *p<0.05, **p<0.01, ***p<0.001.

For 13 genes, a comparison of expression in human tumours and normal cerebellum was also possible (using data from Cho, Tsherniak et al. [35]) and a total of 10 genes show significantly different expression between cerebellum and either SHH subgroup tumours alone or all tumours (Additional file 5: Table S3), consistent with dysregulation of expression during tumorigenesis. Furthermore, the direction of expression change observed is generally consistent with the predicted mode of action of each CIS. For instance, expression levels of *FGF13*, *NFIB*, *TEAD1* and *TGIF2* are all increased versus normal cerebellum whilst expression of *MYT1L*, *SFI1*, and *SLIT3* is appropriately reduced (Table S2) in line with the inferred mechanism of action (Table 1).

### CIS genes define a neuronal transcription factor network in human MBs

**Figure 3:**
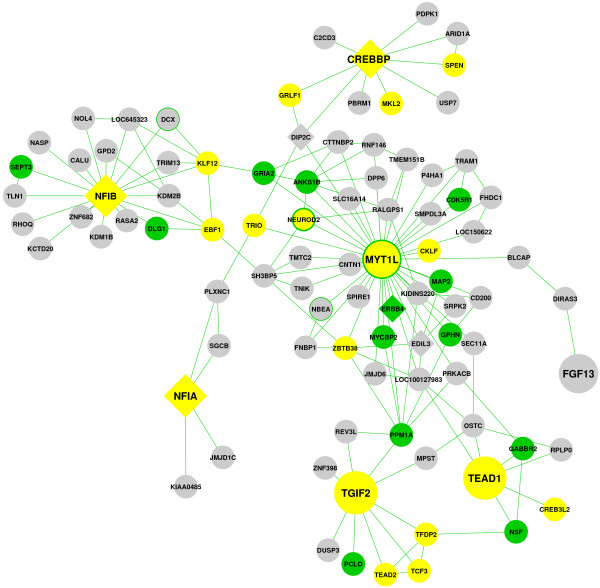
**A neuronally enriched transcription factor network is defined by MB CIS genes.** MB CIS genes (enlarged) linked via nearest neighbours are shown. The topological arrangement of connected CIS nodes is highly significant (p<0.006) as the average minimum path distance between the 7 CISs is lower than in 994/1000 randomly resampled networks. All network edges are supported with a bootstrapped p-value of <1x10^-8^. Genes identified as biallelically mutated MB CISs by Wu et al. [30] are shown as diamonds. Genes with transcription regulator activity (GO ID 30528) are shown in yellow. Genes with enriched ontologies associated with differentiated neurons (GO IDs 45202, 44456, 45211, 43005, 43198) are shown in green. Other genes with a known role in neuronal development are shown with a green border. For details of network construction, see Additional file 7. For details of enriched ontologies see Additional file 6: Table S4.

The significant enrichment for transcription factor (TF) activity within the MB CIS genes raised the possibility that they could be present within co-ordinated signalling or developmental pathways. ARACNE [40,41] is a method which uses gene-gene co-regulation measures, and elimination of indirect relationships, to infer TF-target interactions within expression data. It has successfully been used to identify novel oncogenes in expression datasets from glioma [42] and acute lymphoblastic leukaemia [43]. We used ARACNE to infer regulatory networks within publicly available MB gene expression data ([38,39] see Methods). Strikingly, seven CIS genes, including four of the five CISs with the highest GKC p-values, were linked within a single network either directly or via nearest neighbours (Figure 3). This cluster of CIS nodes is highly significant (p=0.006 using 1000 randomly re-sampled networks) and consists of 6 genes with transcription factor/cofactor activity (*CREBBP, MYT1L*, *NFIA*, *NFIB*, *TEAD1* and *TGIF2*) and one neuronal growth factor (*FGF13*). Gene ontology analysis (see Methods) established that the extended network is enriched both for transcription factors/regulators (p=0.0026/0.0035, Additional file 6: Table S4, yellow in Figure 3) and for genes with ontologies relating to cellular components of differentiated neurons (p=0.019-0.0042, Additional file 6: Table S4, green in Figure 3). This suggests that the network consists primarily of neuronal transcription factors and their targets.

**Figure 4:**
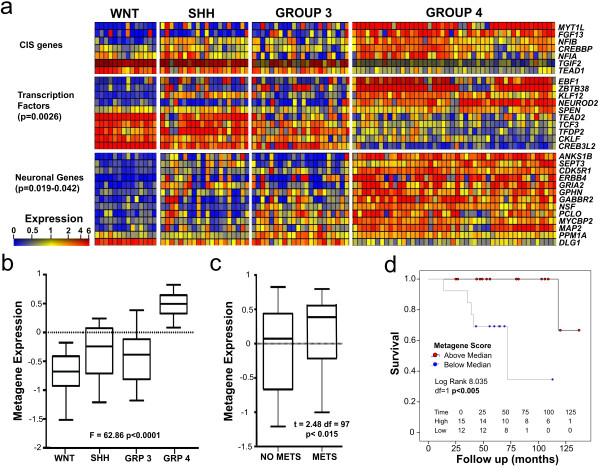
**Network activity correlates with tumour subgroup, metastasis and survival. a.** Gene expression heatmaps of CIS genes and network genes with enriched ontologies in all 4 clinicogenetic subgroups are shown. Where more than one probe per gene was present in the network, the probe with the highest mean expression level across all samples is shown. **b. **Box and whisker plot showing log of metagene expression in MB clinicogenetic subgroups. ANOVA p-value is shown. Boxes represent the 25^th^ to 75^th ^percentile, with median values shown as solid lines. Whiskers represent 95^th ^percentile, and outliers are shown individually. **c. **Log of metagene expression in tumours with metastases and those without. T-test p-value is shown. **d. **Kaplan Meier curve of SHH subgroup tumours relative to Metagene score.

To investigate the relative activity of genes within these enriched ontologies in human tumours, expression heatmaps of CIS genes, transcription factors, and neuronal genes within the network were generated (Figure 4a). There are clear expression differences between clinicogenetic subgroups, consistent both with the ANOVA analysis of CIS gene expression across subgroups (Table 2) and with the presence of genes previously shown to be highly expressed in Group 3 and 4 tumours (e.g. *NEUROD2*, *GABBR2*[38]). However, most striking are the neuronal genes which include neurotransmitter receptors and synaptic scaffold/matrix proteins, the vast majority of which show low expression in the SHH and WNT tumours.

Four of the seven networked CIS genes have recently been identified as MB CISs in a tissue-specific SB mutagenesis screen of primary tumours generated using the *Ptch* model (*Crebbp*, *Nfia*, *Nfib* and *Tead1*[30]), and orthologs of a further three genes within our network (*Dip2c*, *Edil3* and *Erbb4*) were identified as MB CISs in the same screen. Strikingly, with the exception of *Tead1*, both alleles of all of these genes were targeted by inserts in primary tumours in this screen [30], indicative of key tumour promoting events. We therefore analysed the distribution of all 17 biallelic events identified in this tissue-specific study and found them to be significantly enriched within our network (6/90, 7%) compared to outside our network (11/5823, 0.19%; Fisher’s Exact Test p <0.00001). This provides evidence that the TF network identified here was also targeted in an independent SB screen.

### Network activity correlates with advanced disease and survival in SHH tumours

To assess network activity within human tumour datasets further, we generated a single “metagene” metric to summarise the expression of CIS network genes. The expression of each gene was signed according to direction of correlation with other CIS genes, such that a single score reflected the unified action of all genes (see Additional file 7). As a result, the expression of genes where CISs are inferred to cause loss of function was positively correlated with metagene score (e.g. *MYT1L*), whereas the expression of genes where CISs are inferred to cause gain of function (e.g. *TGIF2*, Additional file 2: Figure S1) was negatively correlated. Metagene activity was then investigated with respect to MB subgroups and clinical features. As expected from the expression heatmaps (Figure 4a), the activity of the network metagene differs significantly between MB clinicogenetic subgroups (F = 62.8 p<0.0001 Figure 4b), with the highest network activity being observed in Group 4 tumours. Metagene activity is also higher in tumours presenting with metastatic disease when compared to those that do not (bootstrapped t = 2.388; p<0.013 Figure 4c). This is likely to reflect the high metagene expression in Group 4 tumours where metastases are frequently observed. A subgroup specific analysis of available expression data which has associated survival information [35], however, showed that network activity correlates significantly with survival in SHH subgroup tumours (log-rank 8.03; p <0.005, Figure 4d), but not in other subgroups (data not shown).

### Microarray expression analysis of mutagenised tumours identifies *Igf2* as a key network associated gene

**Figure 5:**
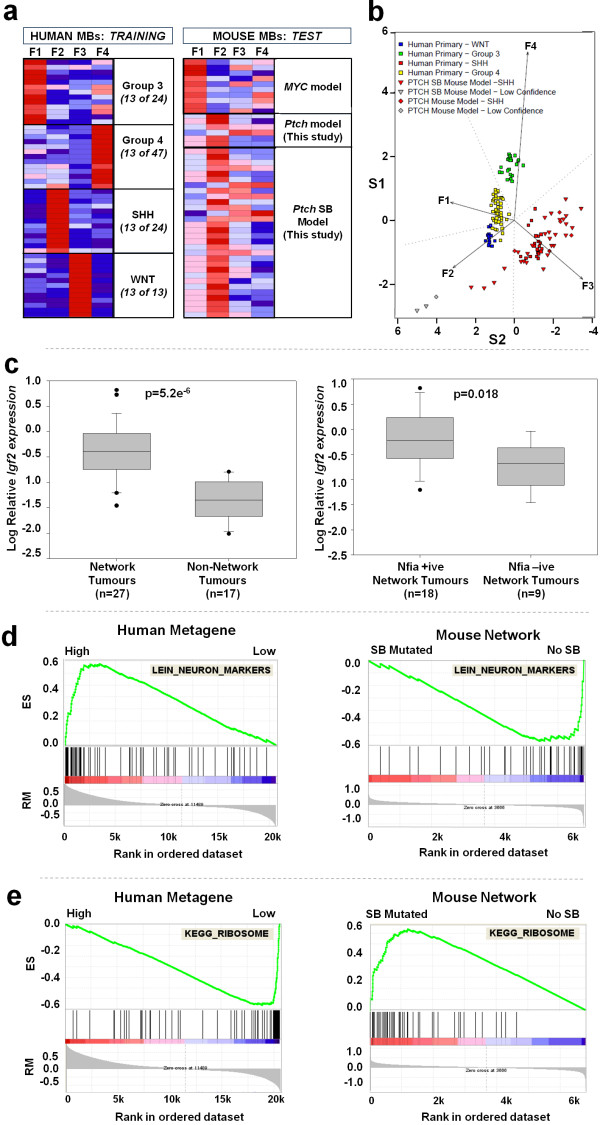
**Ptch**^**+/- **^**MBs are SHH subgroup tumours and *Igf2 *is a key network associated gene. a-b.** Projection of four sub-group specific metagenes derived from 108 human primary MB expression profiles (Training Set) onto MB mouse model expression profiles (Test Set) using Non-negative Matrix Factorisation (NMF). **a.** Heatmaps showing expression of metagenes across human tumours (training; left panel) and expression of metagenes across murine tumours (test; right panel). **b.** Pseudo-plot of NMF projections coloured according to confident MB sub-group classification of murine tumours using metagene expression values. This unsupervised analysis shows that the majority of *Ptch* mouse model samples recapitulate the expression profiles of primary SHH MB. **c.** Box and whisker plots showing relative *Igf2* expression assayed by Real Time PCR (for details, see Methods): Left Panel - tumours with inserts in 1 or more network CIS gene versus tumours with no inserts; Right Panel - *Igf2* expression in tumours with 1 or more insertions in *Nfia* versus all other tumours with inserts in at least 1 network CIS gene. Boxes represent the 25^th^ to 75^th^ percentile, with median values shown as solid lines. Whiskers represent 95^th^ percentile, and outliers are shown individually. **d-e.** GSEA plots for human tumours ranked by metagene score (left panels) and mouse tumours ranked by fold change of expression in tumours with network hits compared to predisposition control MBs not exposed to mutagenesis (right panels). ES-Enrichment Score, RM-Ranked list Metric. **d.** Lein_Neuron_Markers. Human NES=2.15 p<0.001, Mouse NES=-2.24 p<0.001. **e.** KEGG_Ribosome. Human NES=-3.00 p<0.001, Mouse NES=2.59 p<0.001.

**Figure 6:**
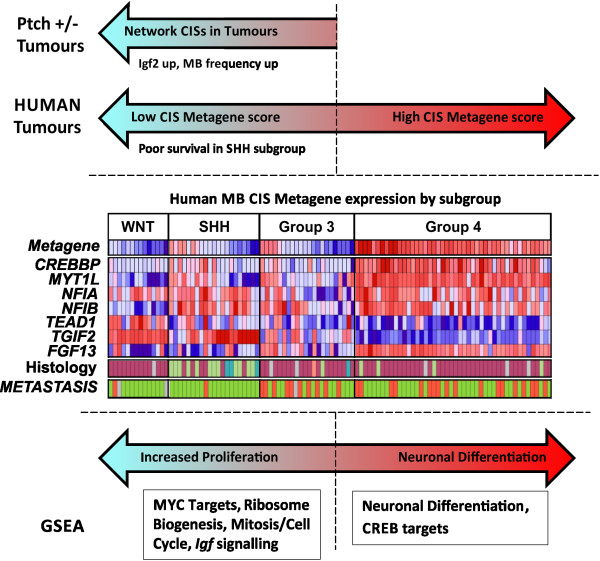
**Integrated summary of metagene expression, associated clinical features, and GSEA.** The schematic relationship between mouse and human tumours in terms of network genes is shown in the top panel. Expression heatmaps showing the network metagene and CIS genes in 108 MBs by subgroup [38,39], together with the incidence of metastatic disease, are shown in the middle panel. For all genes, data from the most highly expressed Affymetrix probe are shown. In the metastases track, red = presence, green = absence, grey = no data. In the histological subtype track, magenta = classic, green = desmoplasia, blue = LCA. A schematic of key processes relevant to tumour biology identified by GSEA is shown in the bottom panel. For details of GSEA, see text and Additional file 9: Table S6.

To identify specific genes whose expression might be altered by network mutations, we generated murine expression data using Illumina bead arrays (see Methods) from 30 SB induced tumours, 6 non-mutagenised tumours from *Ptch*+/- animals, and 6 normal cerebella. We first validated the mutagenised murine model in terms of gene expression as follows: Four metagenes were generated from human tumour expression data using NMF (see Methods) to define the four MB biological subgroups. These metagenes were then projected across the mouse tumour data using all available orthologous probes (Figure 5a), and the subgroup identity of the mouse tumours was then tested with a Support Vector Machine (SVM) using the metagene scores for the human data as the training set and the mouse tumours as the test set. All human tumours were trained correctly with zero errors. The mouse SB tumours were also correctly predicted to be SHH tumours in 29/30 (96%) of cases (Figure 5b) and 6/6 (100%) of non-transposon PTCH MB controls, establishing that gene expression in SB induced mouse tumours is similar to expression in human SHH tumours.

We then looked for genes differentially expressed between tumours with hits in CIS network genes and tumours with no hits in these genes. This identified the Insulin-like growth factor 2 (*Igf2)* as the most differentially expressed gene with a mean fold change of 3.58 (p=0.002, Additional file 8: Table S5). To validate the association between network insertions and *Igf2* expression, we quantified *Igf2* expression in all of our SB-induced MB tumours for which RNA was available using Real Time PCR (See Methods). This confirmed that *Igf2* was expressed at a significantly higher level in tumours with one or more insertions in a network CIS gene, than in tumours with no insertion in a network CIS gene (p<0.0001, Figure 5c). Furthermore, we also established that tumours with hits in *Nfia*, the CIS gene most frequently affected by SB insertion, expressed *Igf2* at higher levels than network tumours with no insert in *Nfia* (Figure 5c).

### CIS related Network activity is associated with proliferation and reduced differentiation

Finally, to gain insight into biological processes which may be affected by the network, we also performed a Gene Set Enrichment Analysis (GSEA) which uses gene ranking to test for enrichment of predefined genesets [44]. This was performed both in mouse tumours (ranking genes by fold change of expression in SB-induced tumours with network hits compared to *Ptch+/-* MBs not exposed to mutagenesis) and in primary human MBs (ranking by association with metagene score).

GSEA revealed a broad picture of increased cell proliferation and reduced differentiation associated with network hits in mice and low network metagene activity in human tumours (the concordant gene expression pattern), as demonstrated by significant enrichment of multiple genesets (see Additional file 9: Table S6). For instance, genesets indicative of neuronal differentiation, such as “Cahoy_Neuronal” and “Lein_Neuron_Markers”, are significantly enriched in human MBs with high metagene expression and in MBs from murine *PTCH* controls with no transposition (e.g. Figure 5d). A similar effect is seen for genesets describing genes containing CREB and cAMP responsive elements (e.g. Additional file 10: Figure S3A). In contrast, genesets denoting proliferation and elevated cell growth are significantly enriched in human MB with low network metagene activity and mouse *PTCH* MBs with CIS network hits. These include genesets linked to mitosis and cell cycle, MYC targets (Additional file 10: Figure S3B), and ribosome biogenesis (Figure 5e).

Consistent with increased *Igf2* expression in MBs from mice with network hits, enrichment of IGF-related genesets is also observed in human and mouse tumours; e.g. enrichment of Pacher_Targets_of_*IGF1*_and_*IGF2*_up in mouse tumours with network hits (Additional file 10: Figure S3C), and of Boudoukha_Bound_by_*IGF2BP2* in human tumours with a low Metagene score (Additional file 10: Figure S3D). In addition, the genesets Lee_Targets_of *PTCH1*_and_*SUFU “*_up” and “_dn”, indicative of SHH dependent murine tumorigenesis [45], show consistent differential enrichment; upregulated targets are enriched in mouse network tumours and human tumours with a low Metagene score (Additional file 10: Figure S3E), and downregulated targets are enriched in mouse tumours with no mutagenesis and human tumours with a high Metagene score (Additional file 10: Figure S3F). Collectively, the broad concordance of human and murine datasets indicates conservation of CIS mutational function across species, and substantiates the concerted action of CIS network genes as a tumourigenic process promoting cell proliferation and inhibiting neuronal differentiation.

## Discussion

We have shown that whole body SB mutagenesis of *Ptch*+/- mice significantly enhances MB frequency without affecting latency, and does not induce these tumours in wild type mice. The majority of candidate genes identified have either been implicated in neuronal development, differentiation and/or migration, have been linked to SHH signalling, or have been shown to be mutated in SHH subgroup tumours in humans. Furthermore, we found that one gene identified from our screen, *MYT1L*, is of prognostic value within a multivariate analysis of human MB survival data. These genes, therefore, warrant individual assessment as potential therapeutic targets and/or biomarkers for the improved stratification and treatment of medulloblastoma.

Notably, 7 of the genes (6 transcription factors/cofactors and 1 growth factor) show significant associations with each other within a novel MB expression network, implying a previously unidentified functional relationship which does not map to established canonical pathways. This network is enriched for transcriptional regulators and genes with neuronal ontologies, and links genes with roles in stem cell/neuron migration, neurite growth and neuronal cell cycle progression, to genes which encode structural and functional elements of mature neurons (See Additional file 11: Table S7 for known gene functions). This suggests that the network comprises transcription factors involved in the proliferation and differentiation of cGNPs, the cell of origin for SHH MB [7], and their targets. Interestingly, lineage commitment to cGNP identity is a pre-requisite for SHH tumour development [46,47].

The relationships identified here between murine network activity, metagene activity in human tumour subtypes, and individual CIS gene expression, are summarised in Figure 6. This highlights the variable network activity within SHH tumours and Group 3 tumours, and identifies CIS genes with similar, or wholly divergent, expression patterns relative to the metagene. Of these, *TGIF2* and *MYT1L* are of particular interest as they influence neuronal SHH expression and development, respectively: A conditional *Tgif1*/*Tgif2* double knock-out has recently been shown to reduce *Shh* expression in the developing brain and to recapitulate holoprosencephaly [a human condition caused by *SHH* and *TGIF1* mutations (OMIM# 142945 and 142946)], while the transcriptional repressor *MYT1L* can contribute to the re-programming of human fibroblasts into neurons [48,49]. As neither gene has been implicated in MB development to date, both are prime targets for further investigation.

Importantly, network activity has clinical relevance, as high activity is associated with advanced disease in all tumours and low activity is associated with poor survival specifically in SHH subgroup tumours (Figure 6). These associations appear incongruous, but the former is likely due to the high incidence of metastases in Group 4 tumours where network activity is uniformly high. In contrast, the SHH subgroup-specific association with outcome may reflect clinically important variation in the developmental status of individual tumours, and highlights the potential utility of network activity as a prognostic biomarker for the prediction of outcome within the SHH subgroup.

The GSEA analysis in mouse and human tumours demonstrates very clearly a role for this network in inhibiting neuronal differentiation and promoting cell proliferation. Consistent with this, several common functional pathways were identified in both species of potential relevance to disease. Of these, the MYC and *IGF*-dependent signalling pathways are of particular interest, the latter having recently been highlighted in an independent SB screen [30]. Furthermore, our analysis of gene expression in SB mutagenized mouse tumours identified *Igf2* upregulation as a key output of SB-induced network perturbation. *Igf2* is already known to be required for MB development in the *Ptch*^+/-^ model as no tumours are observed in *Igf2* null; *Ptch*^+/-^ mice [50], over-expression of *Igf2* in *Ptch*^+/-^ mice increases the frequency of MBs generated by *Shh* transfection of cerebellar neural progenitors [51], and at the cellular level *Igf2* acts synergistically with *Shh* to increases murine cGNP cell proliferation 10 fold [52].

The results presented here suggest that network mutations converge to inhibit differentiation and upregulate *Igf2*. This extends the existing model of MB formation in SB mutagenised *Ptch*^+/-^ mice by identifying genes underpinning the upregulation of *Igf2* which leads to the persistence of *Ptch*^+/-^-induced cerebellar proliferative lesions and progression to MB [53]. Consistent with this model, several network CIS genes, or genes which they bind/modulate, have already been implicated in *Igf2* expression or activity, including *Tead1*[54], *Nfia* and *Nfib*[55,56], and *Crebbp*[57]. There is an unmet clinical need for the development of SHH pathway-independent targeted therapies for SHH subgroup tumours, particularly in view of the predicted acquired or intrinsic resistance to current SMO inhibitors [9,10]. The implication of insulin-dependent signalling in human and mouse SHH tumours strongly support its development as a therapeutic target for SHH subgroup tumours.

The application of SB mutagenesis to additional murine MB models [17,18] could identify genes relevant to other tumour subgroups. However, our results contrast sharply with a recent *Math1* driven tissue-restricted screen of a more penetrant *Ptch*^+/-^ model [30] where tumour latency was reduced from 8 to 2.5 months, a high frequency of metastases (80%) was observed, and divergent primary and metastatic insertional mutation signatures were defined. Notably, we did not observe metastases following whole-body mutagenesis in this study. These two models are, therefore, not directly comparable and suggest that penetrance of the tumour predisposition, and the power of the mutagen, are likely to determine the nature of genes identified in future screens.

Finally, this is the first time to our knowledge that mutagenesis data from a murine cancer model have been integrated with human expression networks to explore biological mechanisms of tumourigenesis. The identification of novel and biologically relevant candidate genes linked within a single expression network, the activity of which correlates with disease state and survival within the subgroup of tumours being modelled, illustrates the utility of this cross-species approach. Clarification of the interactions between network genes identified here, their roles in the pathways highlighted by our GSEA analysis, and establishment of their therapeutic relevance will, however, require extensive functional analyses of multiple genes both individually and in concert.

## Conclusions

Here, we have used SB mutagenesis to define a novel neuronal transcription factor network involved in medulloblastoma formation within the *Ptch*^+/-^ model, and provide evidence that disruption of this network upregulates *Igf2*, critical for proliferation of GNPs and tumour formation. Moreover, we have identified rational therapeutic targets for SHH subgroup tumours, alongside prognostic biomarkers for the identification of poor-risk SHH patients, supporting the further development of these findings as a basis for improved and individualised therapy. Our results also suggest that the integration of mutagenesis data and expression network analysis may help to unravel key events in other cancers which disrupt complex developmental programmes, for which murine models are available.

## Methods

### Mice strains

The following strains were used: *B6 Ptch1*^*tm1Mps*^/*J* mice [19]; CBA wild type (Charles River laboratories, Margate, UK); T2Onc line 76 [58] and SB11 Rosa26 [22]. All animal work adhered to UK Home Office guidelines and was performed under Project Licence PLL/60/3621. Animal numbers, together with tumour incidence in each genotype, are given in Additional file 1: Table S1.

### Sample processing, insertion site mapping and CIS identification

Tumours and other abnormal tissue identified upon post-mortem examination were collected for histological examination and DNA/RNA isolation. Insertion sites were identified using splinkerette PCR [21] coupled to GS-FLX amplicon sequencing [59] and sequence reads were mapped to the mouse genome (NCBI37/mm9) as described previously [60]. Common insertion sites (CISs) were identified using Gaussian kernel convolution (GKC) [23]. The raw p-value of each CIS peak was corrected for the total number of CIS peaks on the chromosome to which it maps, with a cut off of p<0.05. Monte Carlo simulation methods [24] were also used for comparative purposes.

### Illumina expression analysis and Real Time PCR

200 ng of each RNA was amplified and biotin labelled using the Illumina TotalPrep RNA amplification kit (Applied Biosystems, Foster City, CA. USA). cRNA size distribution was assessed using an Agilent Bioanalyser. Approximately 750 ng of each cRNA was hybridised to the Illumina Mouse8 Reference Array (Illumina, Essex, UK) according to the manufacturer’s recommended protocols by the Wellcome Trust Clinical Research Facility (Edinburgh, UK). Real Time PCR was performed using the 5’ nuclease assay on the ABI PRISM^™^ 7700 Sequence detector (Perkin-Elmer, Applied Biosystems, Foster City, CA, USA). Oligonucleotides were designed using Primer Express software (v3.0, PE Biosystems, Foster City, CA, USA), and were designed to span an intron to avoid amplification from genomic DNA. Mean Ct values were normalised against the average expression of the endogenous control gene β-Actin. Relative gene expression was calculated by the 2^-ΔΔCt^ method [61] using the control cerebellar RNA which showed the highest expression level as measured by the Illumina microarray data. For primer sequences see Additional file 7.

### Bioinformatic Analyses

Expression profiles comprising 119 Affymetrix HGU133p2 arrays were taken from published studies [38,39]. CEL files were processed using the Bioconductor RMA package [62]. The ARACNE network was constructed using the aracne2 standalone software package according to authors instructions [40]. Ontology analyses were performed using the Bingo 2.44 Cytoscape plug in [63], with Benjamini and Hochberg FDR-corrected hypergeometric tests and the whole annotation set as background. The topological arrangement of 7 network genes was significance tested by calculating the mean shortest path distance to the nearest connecting CIS gene and permuting 10,000 times with 7 randomly selected genes in order to create a null distribution.

GISTIC analyses [37] were performed using the module provided in Genepattern [64], with Affymetrix SNP Chip .CEL files [35,36] being processed using the Aroma package [65] and segmented using the CBS algorithm [66]. GSEA [44] was performed using the standalone package (http://www.broadinstitute.org/gsea/) and genesets were taken from the MsigDB library [67]. NMF (Non-Negative Matrix Factorisation) was performed using a script adapted from [68]. All other statistical tests were performed using R [69]. For further details of procedures and analyses, see Additional file 7.

## Availability of supporting data

Microarray gene expression data from this study have been deposited in the Gene Expression Omnibus as submission GSE43994. Insertion site data in the form of BED files are provided as Additional files 12 and 13.

## Competing interests

The authors declared that they have no competing interests.

## Authors’ contributions

MSJ, SCC, CPFR, and HP conceived the research. MŁ and MSJ performed all animal husbandry. MŁ, HAl-A, HHAl-B, HS and EM performed all molecular biological analysis of tumours apart from the GS-FLX sequencing which was performed by JC. CMB performed pathological analysis and classification of all tumours, ADB performed additional pathological review and classification of liver tumours. MS-K performed all murine survival analyses, LC provided MYCN model tumour DNAs, AGR and DJA performed all statistical analysis of transposon inserts, and DW performed all downstream bioinformatic analyses. MSJ, SCC and DW interpreted the data and wrote the manuscript with input from all authors. All authors approved the final version of the manuscript.

## Supplementary Material

Additional file 1: Table S1 A. Tumours in experimental and control cohorts. Median age of onset and range of onset is given for common tumour types. Genotypes: Experimental – *Ptch*+/-;SB11+/-;T2Onc+/-. Predisposition - *Ptch*+/-;T2Onc+/-. Transposition - SB11+/-;T2Onc+/-. ^1^Parenchymal brain lesions were consistent with gliomas and included one with pseudopalisading necrosis consistent with glioblastoma multiforme. ^2^Morphological and immunohistochemical analysis established that ~50% of haematological malignancies were precursor T-cell lymphoblastic lymphomas/leukaemias, ~5% were confirmed myeloid neoplasms, and the remainder were poorly differentiated haematological neoplasms, mostly of probable myeloid lineage. ^3^RMSs developed most frequently on the hindquarters and lower limbs. ^4^Adenomas showed mild dysplasia with frequent intralesional steatosis, although sufficient atypia and eosinophilic cytoplasmic inclusions were observed in 2 nodules to warrant classification as hepatocellular carcinoma. All but 2 were identified in animals where other malignancies were also present (4 RMS, 10 Haematological, 1 with RMS+Haematological, and 2 unclassified). All animals with liver adenomas were male, a highly significant bias (p<0.0001). ^5^Hydrocephalus has previously been reported in this model [19] B. Transposon Insertions by sample group. N – number of samples analysed. All inserts – total number of inserts after initial mapping and filtering. SSIs removed – Number of Same Site Inserts (present in same dinucleotide in different samples) removed. STIs removed – Number of Same Tumour Inserts (multiple inserts present in same CIS from same sample) removed. ^1^One tumour did not yield high quality DNA and could not be used for CIS identification. For details of filtering, see Additional file 7. Click here for file

Additional file 2: Figure S1 MB T2Onc insertions in *Tgif2*. A. Genomic organisation of *Tgif2* is shown with orientation and location of T2Onc insertions indicated, together with PCR amplicons from tumour genomic DNA using primers specific for T2Onc and Exon 2. An amplicon of the expected size is obtained from each tumour, confirming that all inserts defined by the GS-FLX sequencing are present. B. Identification of chimeric T2Onc/*Tgif2* transcripts. The schematic shows the relative position of primers within T2Onc and Exons 1 and 2 of *Tgif2*, together with PCR amplicons obtained from tumour cDNA templates. Top panel – primers E1 and E2. Tumours with inserts show reduced intensity of the expected E1-E2 transcript. Middle panel – primers SD to E2. Chimeric T2Onc/*Tgif2* transcripts are observed only in tumours with inserts. Bottom panel – *Gapdh* loading control. Approximate size of expected amplicons is shown in all cases. M – 100 bp ladder, C – no DNA controls. For primer sequences, see Additional file 7 and Keng et al. [27]. Click here for file

Additional file 3: Table S2 Multivariate analysis of human survival data. Models used the data from Cho Tsherniak et al. [35] and incorporated as variables their “high-risk clinical” factor (which incorporates multiple risk factors including the presence of metastases), and the presence of LCA pathology. WNT subgroup tumours were removed as they are known to have a distinct outcome. For *MYT1L*, low expression was retained as a significant multivariate factor (Relative Risk=2.8 p=0.019) alongside LCA (Relative Risk = 2.983 p=0.013). “Clinical Risk Group” was not significant in combination with Low *MYT1L* expression which was preferentially retained in a forward stepwise model. Likewise, for *PTEN*, low expression was significantly associated with increased risk of death (Relative Risk = 2.6 p = 0.031) independent of LCA pathology (Relative Risk = 3.6 p = 0.003). All models have 1 degree of freedom. Click here for file

Additional file 4: Figure S2 Combined data from Cho, Tsherniak et al. [35] and Northcott, Korshunov et al. [36] were analysed. A. Significant overrepresentation of chromosome 10 loss at the *PTEN* locus and B. significant overrepresentation of chromosome 9 and Chromosome 11 gain at the *NFIB* and *TMEM45B* loci respectively. Dotted lines indicate position of centromeres. Upper values in each panel are G-scores, lower values are q-values. C. SNP array data showing localised gain of *TMEM45B*. Approximate cytogenetic positions are given above the schematic, with Megabase position on chromosome 10 given below it. The region of gain, although small, includes additional genes with known roles in cancer (*ETS1*, *FLI1*) and neuronal development (*PRDM10*). Click here for file

Additional file 5: Table S3 Gene Expression in tumours versus normal cerebellum. Comparisons are shown between all tumours and normal cerebellum (left) and between SHH subgroup tumours only and normal cerebellum (right), using data from Cho, Tsherniak et al. [35]. Genes and associated Affymetrix probe IDs are shown. AveExpr - mean log2 expression levels, t – t test statistic, P.Value – Raw p-value, adj.P.Val – p-value adjusted for multiple tests. Click here for file

Additional file 6: Table S4 Cellular Component and Molecular Function Ontologies over-represented in Network. P-value correction was achieved using a Benjamini and Hochberg False Discovery rate of 0.05. Gene frequencies in the network list and genome are shown. Ontologies marked with an asterisk are not significantly enriched when CIS genes are excluded. Click here for file

Additional file 7 Additional details of sample processing, SB insertion site mapping, statistical analyses, and PCR primers. Click here for file

Additional file 8: Table S5 Expression differences in Network v Non-Network tumours. Genes showing the highest average fold change between tumours with 1 or more insert in a Network CIS and tumours with no network insertions are shown, together with Illumina Probe IDs. All p-values are Student t-tests with 28 degrees of freedom. Click here for file

Additional file 9: Table S6 Network Enriched Genesets in primary human MBs and murine tumours. Significantly enriched genesets are shown separately or combined as an intersection between mouse and human results. Associated Nominal p-values corrected for multiple testing, and FDR q-values are also shown. The excel file contains several pages each referring to one of six MSigDb Libraries C1-C6 (see http://www.broadinstitute.org/gsea/msigdb/index.jsp). Human tumours were ranked by metagene score, mouse tumours were ranked by fold change of expression in tumours with network hits compared to predisposition control MBs not exposed to mutagenesis. For details, see Methods. Click here for file

Additional file 10: Figure S3 GSEA plots for human tumours ranked by metagene score (left panels) and mouse tumours ranked by fold change of expression in tumours with network hits compared to predisposition control MBs not exposed to mutagenesis (right panels). ES-Enrichment Score, RM-Ranked list Metric. A. Human NES=1.37 p=0.003; Mouse NES=-1.62 p=0.005. B. Human NES = -2.33, p<0.001; Mouse NES = 1.80, p<0.001. C. Human NES = 1.74, p=0.002; Mouse NES = -1.41, p=0.067. D. Human NES = 1.36, p=0.07; Mouse NES = -1.68, p<0.001. E. Human NES = -2.29, p<0.001; Mouse NES = 1.77, p = 0.002. F. Human NES = 2.19, p<0.001; Mouse NES= - 1.68, p=0.002. For details, see Additional file 9: Table S6. Click here for file

Additional file 11: Table S7 CIS and Network Genes with functions of potential relevance to MB. Genes implicated in neuronal structure, neuronal development, cell cycle, apoptosis or cancer are listed together with relevant citations. A. CIS genes. Network CISs are shown in bold. B. Other transcriptional regulators and neuronal genes in network (from Figure 3 and Additional file 6: Table S4). Click here for file

Additional file 12 SB inserts in medulloblastomas Bed file containing all SB insertion site information from all medulloblastomas analysed, mapped against the NCBI37/mm9 genome build. Click here for file

Additional file 13 SB inserts in controls Bed file containing all SB insertion site information from all control cerebellar samples analysed, mapped against the NCBI37/mm9 genome build. Click here for file
