# Supplementary material for: Identification of a neuronal transcription factor network involved in medulloblastoma development
Source: Acta Neuropathol Commun. 2013 Jul 11;1:35. doi: 10.1186/2051-5960-1-35 (PMC3893591; doi:10.1186/2051-5960-1-35)
Supplement: Additional file 1: Table S1 — A. Tumours in experimental and control cohorts. Median age of onset and range of onset is given for common tumour types. Genotypes: Experimental – Ptch+/-;SB11+/-;T2Onc+/-. Predisposition - Ptch+/-;T2Onc+/-. Transposition - SB11+/-;T2Onc+/-. 1Parenchymal brain lesions were consistent with gliomas and included one with pseudopalisading necrosis consistent with glioblastoma multiforme. 2Morphological and immunohistochemical analysis established that ~50% of haematological malignancies were precursor T-cell lymphoblastic lymphomas/leukaemias, ~5% were confirmed myeloid neoplasms, and the remainder were poorly differentiated haematological neoplasms, mostly of probable myeloid lineage. 3RMSs developed most frequently on the hindquarters and lower limbs. 4Adenomas showed mild dysplasia with frequent intralesional steatosis, although sufficient atypia and eosinophilic cytoplasmic inclusions were observed in 2 nodules to warrant classification as hepatocellular carcinoma. All but 2 were identified in animals where other malignancies were also present (4 RMS, 10 Haematological, 1 with RMS+Haematological, and 2 unclassified). All animals with liver adenomas were male, a highly significant bias (p<0.0001). 5Hydrocephalus has previously been reported in this model [19] B. Transposon Insertions by sample group. N – number of samples analysed. All inserts – total number of inserts after initial mapping and filtering. SSIs removed – Number of Same Site Inserts (present in same dinucleotide in different samples) removed. STIs removed – Number of Same Tumour Inserts (multiple inserts present in same CIS from same sample) removed. 1One tumour did not yield high quality DNA and could not be used for CIS identification. For details of filtering, see Additional file 7. [file 2051-5960-1-35-S1.PDF]

Supplementary Table S1

A. Tumours in experimental and control cohorts

	Genotype					
	Experimental		Predisposition		Transposition	
	N	Median (range)	N	Median (range)	N	Median (range)
**Cohort Number**	243	-	95		120	-
**Medulloblastoma**	42	17.3 (7-27)	4	19 (15-28)	0	-
**Other Brain (1)**	5	29.2 (23-38)	0	-	4	19.5 (12-25)
**Haematological (2)**	51	27.9 (8-52)	0	-	25	34 (13-54)
**Rhabdomyosarcoma (3)**	33	21.3 (14-36)	29	25.5 (8-49)	0	-
**Liver tumours (4)**	19	29 (20-45)	0		2	-
**Other tumours**	2	-	1	-	5	-
**All tumours**	152	-	34	-	36	-
**Hydrocephalus (5)**	9	-	0	-	1	-

B. Transposon Insertions by sample group

**Sample Groups**	**N**	**All Inserts**	**SSIs Removed**	**STIs Removed**	**Final inserts**
**Medulloblastoma(1)**	41	8573	134	79	8360
**Cerebellum Controls**	31	4147	131	35	3981
	**72**	**12720**	**265**	**114**	**12341**
