# Supplementary figures and images for: Identification of a neuronal transcription factor network involved in medulloblastoma development
Source: Acta Neuropathol Commun. 2013 Jul 11;1:35. doi: 10.1186/2051-5960-1-35 (PMC3893591; doi:10.1186/2051-5960-1-35)

# Supplementary Figure S1

## A Genomic

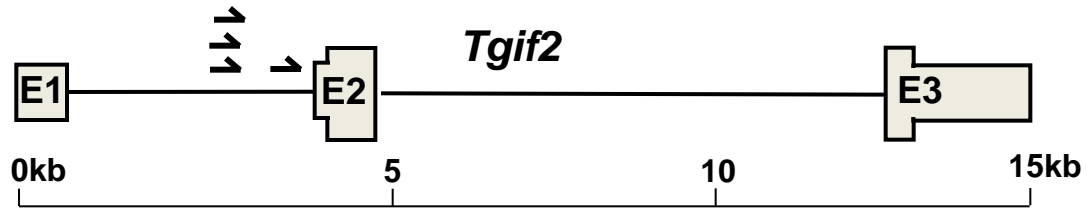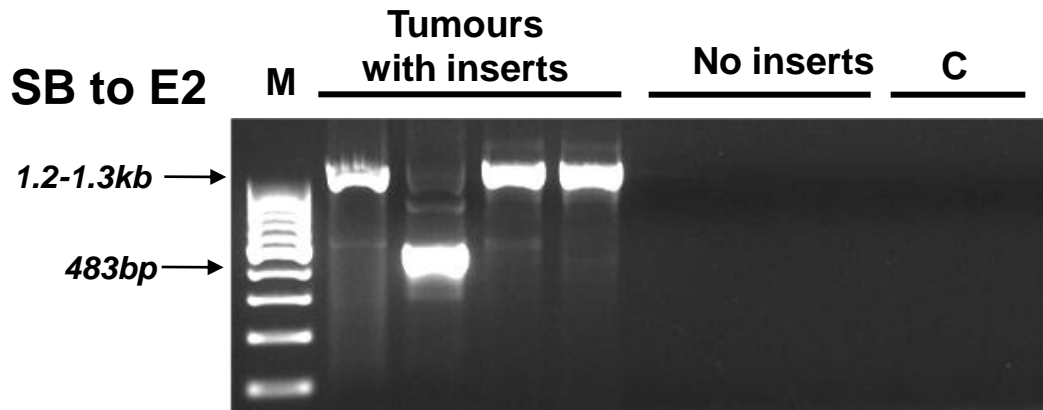

## B Transcripts

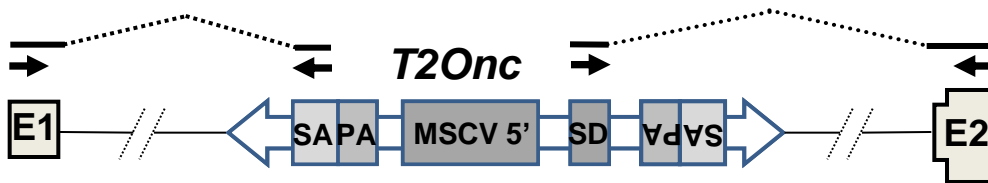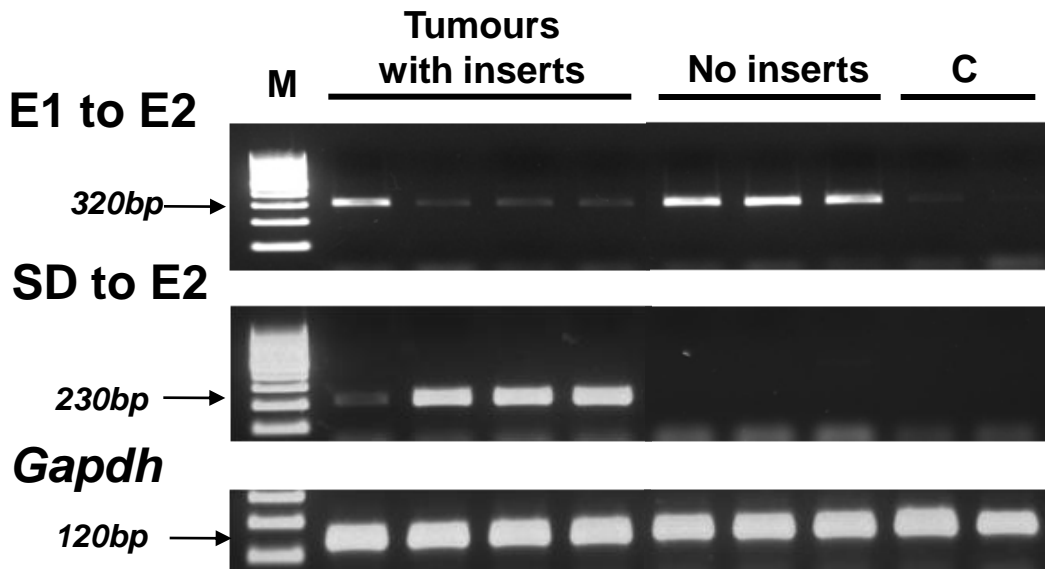

Supplement: Additional file 2: Figure S1 — MB T2Onc insertions in Tgif2. A. Genomic organisation of Tgif2 is shown with orientation and location of T2Onc insertions indicated, together with PCR amplicons from tumour genomic DNA using primers specific for T2Onc and Exon 2. An amplicon of the expected size is obtained from each tumour, confirming that all inserts defined by the GS-FLX sequencing are present. B. Identification of chimeric T2Onc/Tgif2 transcripts. The schematic shows the relative position of primers within T2Onc and Exons 1 and 2 of Tgif2, together with PCR amplicons obtained from tumour cDNA templates. Top panel – primers E1 and E2. Tumours with inserts show reduced intensity of the expected E1-E2 transcript. Middle panel – primers SD to E2. Chimeric T2Onc/Tgif2 transcripts are observed only in tumours with inserts. Bottom panel – Gapdh loading control. Approximate size of expected amplicons is shown in all cases. M – 100 bp ladder, C – no DNA controls. For primer sequences, see Additional file 7 and Keng et al. [27]. [file 2051-5960-1-35-S2.PDF]

Supplementary Figure S2

A. Loss

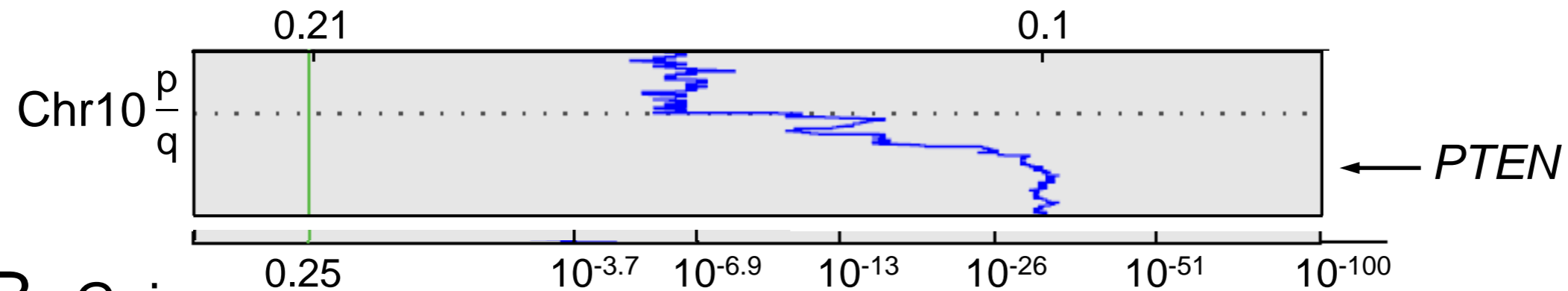

B. Gain

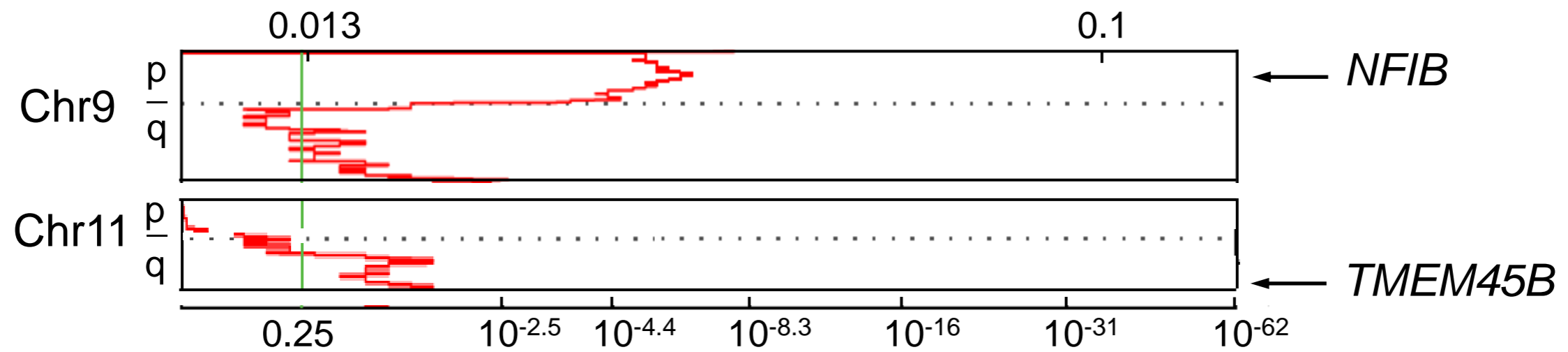

C

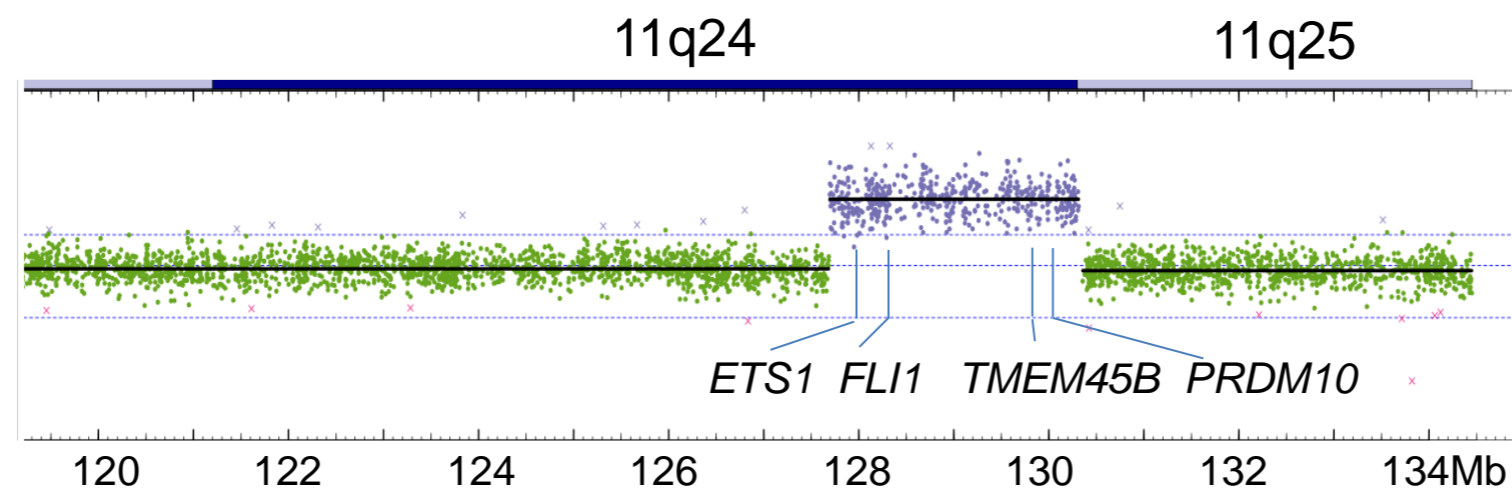

Supplement: Additional file 4: Figure S2 — Combined data from Cho, Tsherniak et al. [35] and Northcott, Korshunov et al. [36] were analysed. A. Significant overrepresentation of chromosome 10 loss at the PTEN locus and B. significant overrepresentation of chromosome 9 and Chromosome 11 gain at the NFIB and TMEM45B loci respectively. Dotted lines indicate position of centromeres. Upper values in each panel are G-scores, lower values are q-values. C. SNP array data showing localised gain of TMEM45B. Approximate cytogenetic positions are given above the schematic, with Megabase position on chromosome 10 given below it. The region of gain, although small, includes additional genes with known roles in cancer (ETS1, FLI1) and neuronal development (PRDM10). [file 2051-5960-1-35-S4.PDF]

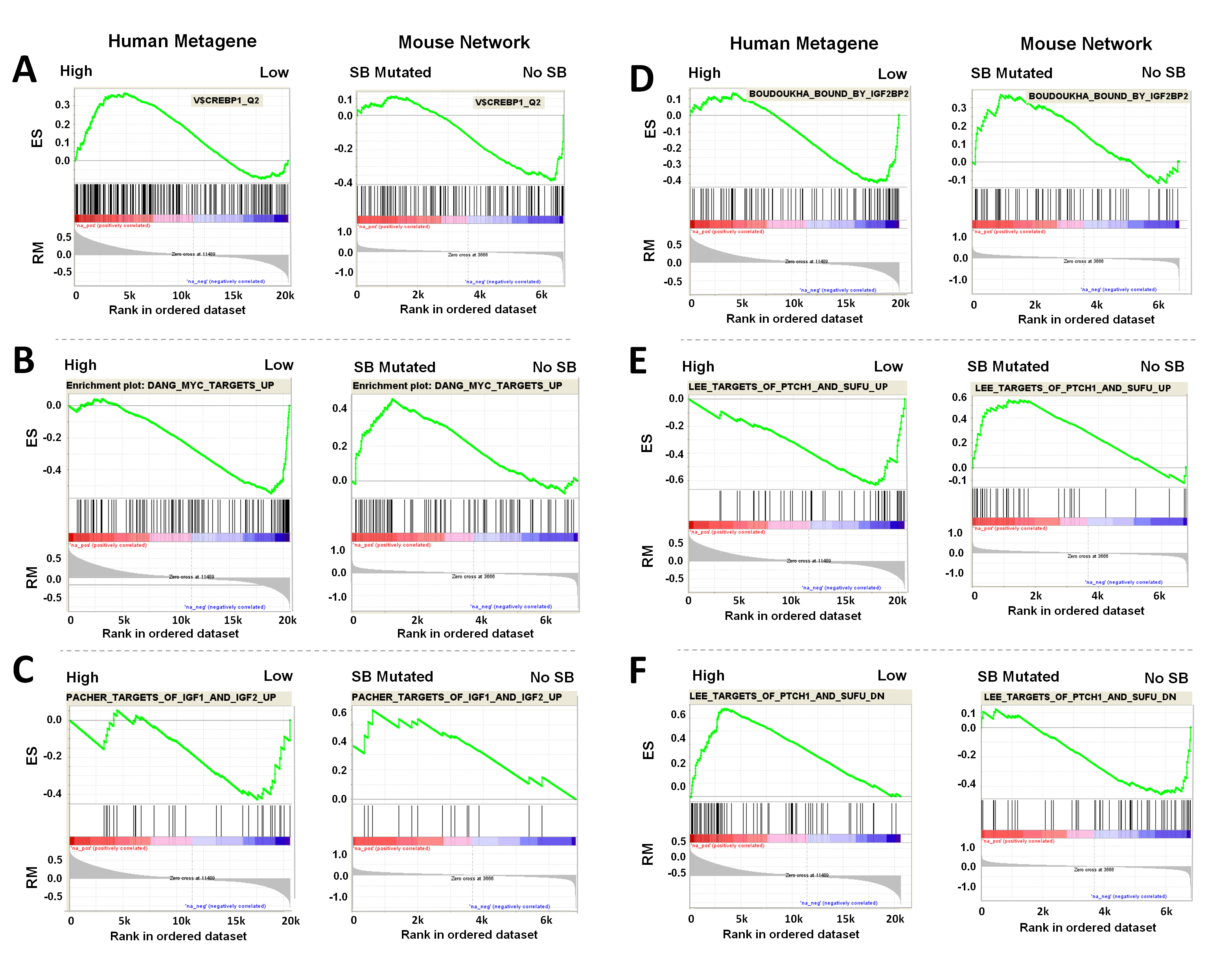

Supplement: Additional file 10: Figure S3 — GSEA plots for human tumours ranked by metagene score (left panels) and mouse tumours ranked by fold change of expression in tumours with network hits compared to predisposition control MBs not exposed to mutagenesis (right panels). ES-Enrichment Score, RM-Ranked list Metric. A. Human NES=1.37 p=0.003; Mouse NES=-1.62 p=0.005. B. Human NES = -2.33, p<0.001; Mouse NES = 1.80, p<0.001. C. Human NES = 1.74, p=0.002; Mouse NES = -1.41, p=0.067. D. Human NES = 1.36, p=0.07; Mouse NES = -1.68, p<0.001. E. Human NES = -2.29, p<0.001; Mouse NES = 1.77, p = 0.002. F. Human NES = 2.19, p<0.001; Mouse NES= - 1.68, p=0.002. For details, see Additional file 9: Table S6. [file 2051-5960-1-35-S10.TIFF]
