# Supplementary material for: Identification of a neuronal transcription factor network involved in medulloblastoma development
Source: Acta Neuropathol Commun. 2013 Jul 11;1:35. doi: 10.1186/2051-5960-1-35 (PMC3893591; doi:10.1186/2051-5960-1-35)
Supplement: Additional file 3: Table S2 — Multivariate analysis of human survival data. Models used the data from Cho Tsherniak et al. [35] and incorporated as variables their “high-risk clinical” factor (which incorporates multiple risk factors including the presence of metastases), and the presence of LCA pathology. WNT subgroup tumours were removed as they are known to have a distinct outcome. For MYT1L, low expression was retained as a significant multivariate factor (Relative Risk=2.8 p=0.019) alongside LCA (Relative Risk = 2.983 p=0.013). “Clinical Risk Group” was not significant in combination with Low MYT1L expression which was preferentially retained in a forward stepwise model. Likewise, for PTEN, low expression was significantly associated with increased risk of death (Relative Risk = 2.6 p = 0.031) independent of LCA pathology (Relative Risk = 3.6 p = 0.003). All models have 1 degree of freedom. [file 2051-5960-1-35-S3.PDF]

Supplementary Table S2: Multivariate Analysis of human survival data.

MYT1L

	B	SE	Wald	p-val	Exp(B)
**Three variable**					
Low MYT1L Expr.	1.314	0.515	6.517	**0.011***	0.269
LCA	0.62	0.694	0.799	0.372	1.859
Clinical Risk group	0.225	0.643	0.122	0.727	1.252
**Two variable (A)**					
Low MYT1L Expr.	1.039	0.444	5.486	**0.019***	0.354
LCA	1.093	0.44	6.16	**0.013***	2.983
**Two variable (B)**					
Low MYT1L Expr.	1.344	0.512	6.893	**0.009***	0.261
Clinical Risk group	0.611	0.426	2.06	0.151	1.843
**Single Variable**					
Low MYT1L Expr.	1.173	0.435	7.253	**0.007****	0.31
LCA	1.295	0.434	8.884	**0.003****	3.651
Clinical Risk group	0.826	0.421	3.845	0.05	2.283

PTEN

	B	SE	Wald	p-val	Exp(B)
**Three variable**					
Low PTEN Expr.	-0.173	0.647	0.072	0.789	1.189
LCA	0.965	0.698	1.91	0.167	2.625
Clinical Risk group	-0.745	0.459	2.634	0.105	0.475
**Two variable (A)**					
Low PTEN Expr.	0.954	0.443	4.63	**0.031***	0.385
LCA	1.296	0.435	8.87	**0.003****	3.654
**Two variable (B)**					
Low PTEN Expr.	0.644	0.457	1.99	0.158	0.525
Clinical Risk group	0.75	0.424	3.137	0.077	2.118
**Single Variable**					
Low PTEN Expr.	0.953	0.443	4.633	**0.031***	0.385
LCA	1.295	0.434	8.884	**0.003****	3.651
Clinical Risk group	0.826	0.421	3.845	0.05	2.283

***p<0.05 **p<0.01**
