# Supplementary material for: Identification of a neuronal transcription factor network involved in medulloblastoma development
Source: Acta Neuropathol Commun. 2013 Jul 11;1:35. doi: 10.1186/2051-5960-1-35 (PMC3893591; doi:10.1186/2051-5960-1-35)
Supplement: Additional file 5: Table S3 — Gene Expression in tumours versus normal cerebellum. Comparisons are shown between all tumours and normal cerebellum (left) and between SHH subgroup tumours only and normal cerebellum (right), using data from Cho, Tsherniak et al. [35]. Genes and associated Affymetrix probe IDs are shown. AveExpr - mean log2 expression levels, t – t test statistic, P.Value – Raw p-value, adj.P.Val – p-value adjusted for multiple tests. [file 2051-5960-1-35-S5.PDF]

Supplementary Table S3

Gene	Affymetrix ID	All Tumours v Cerebellum					SHH Tumours v Cerebellum				
		AveExpr	t	P.Value	adj.P.Val	sig	AveExpr	t	P.Value	adj.P.Val	sig
ANKRD5	220144_s_at	3.194	1.120	0.264	0.444		3.232	1.290	0.202	0.340	
ATXN2	202622_s_at	8.094	-1.558	0.121	0.247		7.932	-3.982	<0.001	0.001	***
CREBBP	202160_at	10.170	4.394	<0.001	<0.001	***	10.020	4.697	<0.001	<0.001	***
DSCR3	203635_at	5.112	1.136	0.257	0.436		5.278	2.033	0.046	0.099	
FGF13	205110_s_at	7.449	3.842	<0.001	0.001	***	5.496	2.305	0.025	0.057	
ITGBL1	214927_at	5.849	-0.624	0.533	0.684		7.958	2.309	0.024	0.057	
MYT1L	210016_at	10.920	-1.791	0.075	0.168		9.940	-4.042	<0.001	0.001	***
NFIB	209290_s_at	13.709	3.923	<0.001	0.001	***	13.746	10.487	<0.001	<0.001	***
PTEN	211711_s_at	8.112	2.852	0.005	0.016	*	8.108	2.761	0.008	0.020	*
SFI1	36545_s_at	5.852	-4.662	<0.001	<0.001	***	6.289	-3.973	<0.001	0.001	***
SLIT3	203813_s_at	2.738	-11.073	<0.001	<0.001	***	2.913	-13.295	<0.001	<0.001	***
TEAD1	214600_at	6.151	2.918	0.004	0.013	*	6.510	6.657	<0.001	<0.001	***
TGIF2	216262_s_at	4.614	4.179	<0.001	<0.001	***	5.989	13.738	<0.001	<0.001	***

***p<0.05   **p<0.01   ***p<0.001**
