# Supplementary material for: Identification of a neuronal transcription factor network involved in medulloblastoma development
Source: Acta Neuropathol Commun. 2013 Jul 11;1:35. doi: 10.1186/2051-5960-1-35 (PMC3893591; doi:10.1186/2051-5960-1-35)
Supplement: Additional file 7 — Additional details of sample processing, SB insertion site mapping, statistical analyses, and PCR primers. [file 2051-5960-1-35-S7.PDF]

Lastowska et al.

Identification of a neuronal transcription factor network involved in medulloblastoma development

Supplementary Methods

Murine crosses and husbandry

To minimise effects of genetic background, all transgenic lines were first back-crossed to CBA for 3 generations. *Ptch*$^{+/-}$;T2Onc$^{+/-}$ double transgenic females were then crossed to *SB11*$^{+/-}$ females to generate both triple transgenic experimental, and double transgenic control, genotypes. *Ptch*$^{+/-}$;T2Onc$^{+/+}$ animals were also crossed to SB11$^{+/+}$ transgenic animals to increase the recovery of triple transgenics. Animals were monitored a minimum of 3 times per week, and humanely culled at the first sign of disease, with the exception of animals with slow growing rhabdomyosarcomas (RMSs) which were euthanized only when additional signs of disease presented or when the tumour impaired movement.

Sample processing

Tumours and other abnormal tissue identified upon post mortem were divided into two equal sections, one snap frozen for DNA/RNA isolation, the other fixed in 10% neutral buffered formalin at room temperature for 24-48 hours before being transferred to 70% ethanol and processed to paraffin blocks. For microscopic examination, 5μm sections were stained with haematoxylin and eosin (H&E). DNA was isolated using a Qiagen Bio Robot EZ1 machine (Qiagen, Hilden, Germany) following digestion with proteinase K, quantified using the NanoDrop ND-1000 spectrophotometer (NanoDrop, Rockland, DE), and molecular weight was assessed by electrophoresis. All RNAs were isolated using the Qiagen RNeasy mini kit, with the exception of brain tissue which was isolated using the Qiagen RNeasy Lipid Tissue mini kit (Qiagen Hilden, Germany). The quantity and quality of all RNAs were assessed using the Agilent 2100 Bioanalyser (Agilent Technologies, Santa Clara, CA). All RNAs used had an RNA Integrity Number (RIN) of 8.0 or above, with the exceptions of MB524 (7.7) and MB244 (6.7).

Insertion sites within 41 MBs were analysed, each from independent animals. To control for potential transposon or tissue-specific insertion site preferences of the *T2Onc* element, 31 cerebella samples from 5-7 week old transposition control animals were also analysed. Insertion sites within tumour and control tissues were amplified using splinkerette PCR directed to both termini of the *T2Onc* transposon as previously described (Collier, Carlson et al. 2005) except that barcoded GS-FLX sequencing primers were introduced during the 2nd PCR (Uren, Mikkers et al. 2009). Samples were then pooled and sequenced using either the GS-FLX LR70 or Titanium (XLR70) protocols (Roche, Basel, Switzerland). A total of 3 LR70 and 5 Titanium runs were performed according to manufacturer's recommendations by NewGene (Newcastle upon Tyne, UK), with a maximum of 48 samples per LR70 lane, and 120 samples per Titanium lane.

Insertion site mapping and statistical analysis.

Sequence reads were filtered and mapped to the mouse genome (NCBI37/mm9) to define insertion sites as described previously (March, Rust et al. 2011). Common insertion sites (CISs) were identified using Gaussian kernel convolution (GKC) as this method allows CISs identified at different scales (kernel widths) to be compared (de Ridder, Uren et al. 2006). The raw p-value of each CIS peak was corrected for the total number of CIS peaks on the chromosome to which it maps, with a cut off of $p<0.05$. Monte Carlo simulation methods (Starr, Allaei et al. 2009) were also used for comparative purposes. To exclude local hopping events from the *T2Onc* transgenic array which maps to chromosome 1 (Collier, Carlson et al. 2005) CISs on this chromosome were not analysed. Due to the high incidence of SB-induced haematological disease, MB and adenoma CISs which were also identified as CISs within haematological tumours are not presented unless otherwise stated.

A small number of CISs in all datasets included two or more insertions into the same dinucleotide in independent tumours. To safeguard against possible low level PCR contamination (Starr, Allaei et al. 2009) each insertion site was counted only once for CIS analysis, the tumour with the highest number of reads being arbitrarily scored as +ve for the insert. In addition, a small number of CISs in both experimental and control datasets were dominated by multiple insertions from a single sample. Each CIS exhibiting this pattern was observed in only one dataset, suggesting that the deep sequencing approach was detecting multiple secondary local hopping events. An example is shown below.

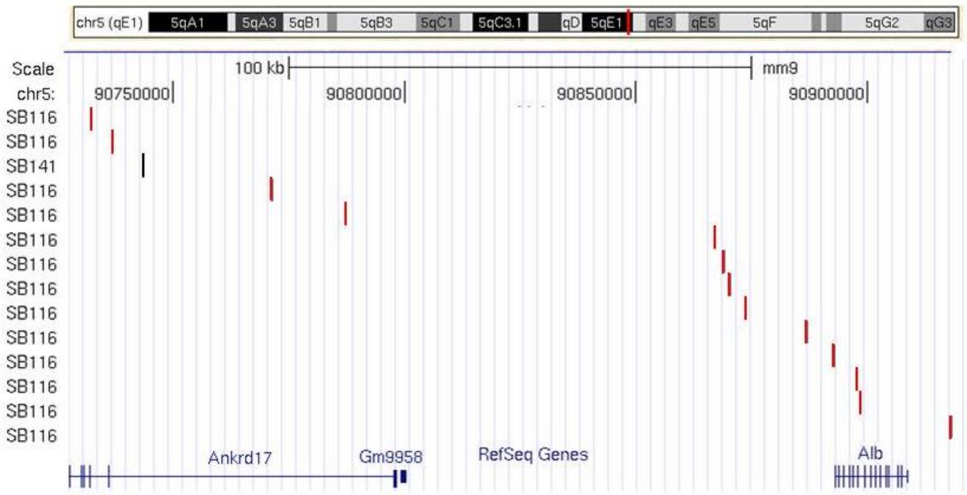

Example of CIS dominated by inserts within a single tumour. Image adapted from UCSC browser showing T2Onc insert positions within a CIS from Cerebellum Controls. Of the 14 inserts, 13 are from a single tumour (SB116 – inserts in red).

To minimise the impact of this phenomenon on CIS calling, a maximum of two inserts per sample within a 15kb kernel width were included in any CIS, and CISs with 50% or more insertions derived from a single sample or defined by less than three samples were excluded from further analyses.

Details of bioinformatic analyses

ARACNE (http://wiki.c2b2.columbia.edu/califanolab/index.php/Software/ARACNE) analysis was performed with adaptive partitioning, an initial p-value cutoff of 1e-7, and a Data Processing Inequality of 0.01. The network was further refined by 100 bootstrap replicates at a significance cutoff of 1e-8. The expression results were filtered so that only genes expressed in >10% of individuals, and which varied three fold with an absolute difference of 500 intensity units from highest to lowest expressing individuals after excluding outliers, were included in the analysis.

Metagene analysis was performed using the average scaled, centred expression of each network gene. Gene expression was signed according to the correlation matrix of the 7 genes in order to best reflect the inferred mechanism of action of the CIS and best preserve a concerted direction to the metagene score. Consequently the sign of the log expression of two genes (*TEAD1* and *TGIF2*) was reversed such that they ultimately negatively correlated with metagene expression. For NMF, metagenes describing all four MB subgroups were derived from primary human tumour profiles. These were then projected onto the mouse arrays mapping by ortholog and used as the basis of support vector machine (SVM) classification, whereby the learning machine was trained on Human primary tumours and tested on Mouse primary tumours. For GSEA, genes were pre-ranked in primary human MBs according to correlation with the network metagene score, and in murine MBs by fold change of expression in SB-induced tumours with network hits compared to *Ptch+/-* control MBs not exposed to mutagenesis.

PCR primers

PCR primers used for insert validation were as follows: *Tgif2*_X1_F GTGTCAGACCCGGCAGGTAC, *Tgif2*_X2_R TTCTCCTGCTCTGAGGGGTA, SD+F AACGCCCGCGAGGAT. All SB and T2Onc primers are from Keng et al. (2009). Real Time PCR primers and probes used were as follows: *Igf2*; For-CGGACTGTCTCCAGGTGTC, Rev-GTCCGAGAGGGACGTGTCTA, used at a concentration of 300nM, Probe-GGAAGAACTTGCCCACGGGG. β-Actin; For-GGTCATCACTATTGGCAACG, Rev-ACGGATGTCAACGTCACACT, used at a concentration of, Probe-AGCGGTTCCGATGCCCTGAG. *Gapdh*; For-AGCGAGACCCCACTAACATC, Rev-GGAGATGATGACCCTTTTGG, used at a concentration of 300nM, Probe-ACCACCATGGAGAAGGCCGG.
