# Supplementary material for: Identification of a neuronal transcription factor network involved in medulloblastoma development
Source: Acta Neuropathol Commun. 2013 Jul 11;1:35. doi: 10.1186/2051-5960-1-35 (PMC3893591; doi:10.1186/2051-5960-1-35)
Supplement: Additional file 8: Table S5 — Expression differences in Network v Non-Network tumours. Genes showing the highest average fold change between tumours with 1 or more insert in a Network CIS and tumours with no network insertions are shown, together with Illumina Probe IDs. All p-values are Student t-tests with 28 degrees of freedom. [file 2051-5960-1-35-S8.PDF]

Supplementary Table S5

Gene Expression Difference between Tumours with and without Network Hits

Illumina PROBE_ID	GENE	Mean Fold Change in Expression : Network vs non-Network	p-value
ILMN_2597769	*Igf2*	3.58	0.002
ILMN_1255871	*Loxl1*	1.64	0.029
ILMN_2757232	*Aqp4*	1.64	0.037
ILMN_1242456	*Kank1*	0.66	0.002
ILMN_2642012	*LOC100046457*	0.65	0.007
ILMN_2731265	*Hsd11b2*	0.65	0.026
ILMN_1252076	*Lyz2*	0.64	0.040
ILMN_1230157	*Rnd3*	0.61	0.038
