# Supplementary material for: Identification of a neuronal transcription factor network involved in medulloblastoma development
Source: Acta Neuropathol Commun. 2013 Jul 11;1:35. doi: 10.1186/2051-5960-1-35 (PMC3893591; doi:10.1186/2051-5960-1-35)
Supplement: Additional file 11: Table S7 — CIS and Network Genes with functions of potential relevance to MB. Genes implicated in neuronal structure, neuronal development, cell cycle, apoptosis or cancer are listed together with relevant citations. A. CIS genes. Network CISs are shown in bold. B. Other transcriptional regulators and neuronal genes in network (from Figure 3 and Additional file 6: Table S4). [file 2051-5960-1-35-S11.PDF]

Supplementary Table S7. CIS and Network gene functions of potential relevance to MB.

A

Gene	CIS P value	Gene Function/Activity of potential relevance to MB
NFIA	<1E-14	Initiates neuronal differentiation, represses self renewal genes [1], modulates *Igfbp5* transcription [2].
ATXN2	<1E-14	Defects in *Atxn2* cause spinocerebellar ataxia type 2 [3].
TEAD1	3.9E-14	Transactivates the effector of the Hippo pathway *YAP* [4] which is amplified in some SHH MBs [5] and activates *Akt* through *IGF2* [6].
TGIF2	0.0006	Transcriptional repressor [7] and paralog of the neuronal developmental regulator *TGIF* [8], loss of *TGIF*s disrupt SSH signalling [9].
CREBBP	0.0009	Somatically mutated in MB [10], co-activates *EGR1* which induces *IGF2* expression [11].
DSCR3	0.0009	-
PTEN	0.0046	Somatically mutated in MB [10], promotes MB formation in SmoA1+/- model [12], modulates proliferative response to *IGF2* [13].
ITGBL1	0.0062	-
NFIB	0.0071	Required for brain development [14], modulates *Igfbp5* transcription [2].
MYT1L	0.0089	Pan-neural Transcription Factor [15], enhances neuronal reprogramming *in vitro* [16, 17].
ANKRD5	0.0092	-
SLIT3	0.012	Represses neurite outgrowth [18], Expression in brain patterned by SHH [19], tumour suppressor activity [20].
TMEM45B	0.019	-
SFI1	0.021	Implicated in G2/M transition [21].
FGF13	0.026	Regulates neuronal migration [22].
L3MBTL4	0.043	Transcriptional repressor, mutated in breast cancer [23].
ADCY5	0.044	-

B

Gene	Gene Function/Activity of potential relevance to MB
PPM1A	Role in neuroblast migration [24].
MYCBP2	Promotes axon guidance/development [25].
GPHN	Neuron assembly protein [26].
NBEA	Implicated in synapse formation [27].
GRIA2	Glutamate receptor [28].
ANKS1B	Implicated in neurite growth [29].
DCX	Promotes neuronal progenitor cell migration [30].
NSF	Regulates *GABA* receptor trafficking [31].
ERBB4	Role in pro-differentiation signalling in Cerebellar Granule Neurons [32].
CDK5R1	Neuronal *CDK5* activator [33], involved in neuronal migration [34] and suppression of neuronal cell cycle [35].
GABBR2	*GABA* receptor [36].
NEUROD2	Induces neuronal transcription [37].
PCLO	Pre-synaptic matrix protein [38].
MAP2	Differentiation marker, affects dendrite shape [39]
DLG1	Interacts with *PTEN* to negatively regulate myelination [40].
EBF1	Neuronal transcription factor, role in retina development [41].
KLF12	-
ZBTB38	Caspase 3 substrate, negatively regulates apoptosis [42]
TEAD2	Transactivates the effector of the Hippo pathway *YAP* to promote cell growth [4].
TCF3	Retards neural stem cell differentiation [43].
CKLF	Induces neuronal migration [44].
CREB3L2	-
TFDP2	Transcription factor, modulates cell cycle gene expression [45].

1. Piper, M., et al., *NFIA controls telencephalic progenitor cell differentiation through repression of the Notch effector Hes1.* J Neurosci, 2010. **30**(27): p. 9127-39.
2. Perez-Casellas, L.A., et al., *Nuclear factor I transcription factors regulate IGF binding protein 5 gene transcription in human osteoblasts.* Biochim Biophys Acta, 2009. **1789**(2): p. 78-87.
3. Verbeek, D.S. and B.P. van de Warrenburg, *Genetics of the dominant ataxias.* Semin Neurol, 2011. **31**(5): p. 461-9.
4. Chen, L., P.G. Loh, and H. Song, *Structural and functional insights into the TEAD-YAP complex in the Hippo signaling pathway.* Protein Cell, 2010. **1**(12): p. 1073-83.
5. Fernandez, L.A., et al., *YAP1 is amplified and up-regulated in hedgehog-associated medulloblastomas and mediates Sonic hedgehog-driven neural precursor proliferation.* Genes Dev, 2009. **23**(23): p. 2729-41.
6. Fernandez, L.A., et al., *Oncogenic YAP promotes radioresistance and genomic instability in medulloblastoma through IGF2-mediated Akt activation.* Oncogene, 2012. **31**(15): p. 1923-37.
7. Melhuish, T.A., C.M. Gallo, and D. Wotton, *TGIF2 interacts with histone deacetylase 1 and represses transcription.* J Biol Chem, 2001. **276**(34): p. 32109-14.
8. Reich, M., et al., *GenePattern 2.0.* Nat Genet, 2006. **38**(5): p. 500-1.
9. Taniguchi, K., et al., *Loss of Tgif function causes holoprosencephaly by disrupting the SHH signaling pathway.* PLoS Genet, 2012. **8**(2): p. e1002524.
10. Robinson, G., et al., *Novel mutations target distinct subgroups of medulloblastoma.* Nature, 2012.
11. Svaren, J., et al., *EGR1 target genes in prostate carcinoma cells identified by microarray analysis.* J Biol Chem, 2000. **275**(49): p. 38524-31.
12. Castellino, R.C., et al., *Heterozygosity for Pten promotes tumorigenesis in a mouse model of medulloblastoma.* PLoS One, 2010. **5**(5): p. e10849.
13. Church, D.N., et al., *Igf2 ligand dependency of Pten(+/-) developmental and tumour phenotypes in the mouse.* Oncogene, 2011.
14. Steele-Perkins, G., et al., *The transcription factor gene Nfib is essential for both lung maturation and brain development.* Mol Cell Biol, 2005. **25**(2): p. 685-98.
15. Romm, E., et al., *Myt1 family recruits histone deacetylase to regulate neural transcription.* J Neurochem, 2005. **93**(6): p. 1444-53.
16. Pang, Z.P., et al., *Induction of human neuronal cells by defined transcription factors.* Nature, 2011. **476**(7359): p. 220-3.
17. Ambasudhan, R., et al., *Direct reprogramming of adult human fibroblasts to functional neurons under defined conditions.* Cell Stem Cell, 2011. **9**(2): p. 113-8.
18. Lin, L. and O. Isacson, *Axonal growth regulation of fetal and embryonic stem cell-derived dopaminergic neurons by Netrin-1 and Slits.* Stem Cells, 2006. **24**(11): p. 2504-13.
19. Barresi, M.J., et al., *Hedgehog regulated Slit expression determines commissure and glial cell position in the zebrafish forebrain.* Development, 2005. **132**(16): p. 3643-56.
20. Marlow, R., et al., *SLITs suppress tumor growth in vivo by silencing Sdf1/Cxcr4 within breast epithelium.* Cancer Res, 2008. **68**(19): p. 7819-27.
21. Ma, P., et al., *Deletion of SFI1, a novel suppressor of partial Ras-cAMP pathway deficiency in the yeast Saccharomyces cerevisiae, causes G(2) arrest.* Yeast, 1999. **15**(11): p. 1097-109.
22. Wu, Q.F., et al., *Fibroblast growth factor 13 is a microtubule-stabilizing protein regulating neuronal polarization and migration.* Cell, 2012. **149**(7): p. 1549-64.
23. Addou-Klouche, L., et al., *Loss, mutation and deregulation of L3MBTL4 in breast cancers.* Mol Cancer, 2010. **9**: p. 213.
24. Khodosevich, K., P.H. Seeburg, and H. Monyer, *Major signaling pathways in migrating neuroblasts.* Front Mol Neurosci, 2009. **2**: p. 7.
25. Po, M.D., C. Hwang, and M. Zhen, *PHRs: bridging axon guidance, outgrowth and synapse development.* Curr Opin Neurobiol, 2010. **20**(1): p. 100-7.
26. Tretter, V., et al., *Gephyrin, the enigmatic organizer at GABAergic synapses.* Front Cell Neurosci, 2012. **6**: p. 23.

27. Medrihan, L., et al., *Neurobeachin, a protein implicated in membrane protein traffic and autism, is required for the formation and functioning of central synapses.* J Physiol, 2009. **587**(Pt 21): p. 5095-106.
28. Liu, Y., et al., *A single fear-inducing stimulus induces a transcription-dependent switch in synaptic AMPAR phenotype.* Nat Neurosci, 2010. **13**(2): p. 223-31.
29. Shin, J., et al., *Identification of phosphotyrosine binding domain-containing proteins as novel downstream targets of the EphA8 signaling function.* Mol Cell Biol, 2007. **27**(23): p. 8113-26.
30. Toriyama, M., et al., *Phosphorylation of doublecortin by protein kinase A orchestrates microtubule and actin dynamics to promote neuronal progenitor cell migration.* J Biol Chem, 2012. **287**(16): p. 12691-702.
31. Chou, W.H., et al., *GABAA receptor trafficking is regulated by protein kinase C(epsilon) and the N-ethylmaleimide-sensitive factor.* J Neurosci, 2010. **30**(42): p. 13955-65.
32. Xie, F., M. Padival, and R.E. Siegel, *Association of PSD-95 with ErbB4 facilitates neuregulin signaling in cerebellar granule neurons in culture.* J Neurochem, 2007. **100**(1): p. 62-72.
33. Tang, D., et al., *An isoform of the neuronal cyclin-dependent kinase 5 (Cdk5) activator.* J Biol Chem, 1995. **270**(45): p. 26897-903.
34. Moncini, S., et al., *The role of miR-103 and miR-107 in regulation of CDK5R1 expression and in cellular migration.* PLoS One, 2011. **6**(5): p. e20038.
35. Zhang, J., et al., *Cdk5 suppresses the neuronal cell cycle by disrupting the E2F1-DP1 complex.* J Neurosci, 2010. **30**(15): p. 5219-28.
36. Chung, S.H., C.T. Kim, and R. Hawkes, *Compartmentation of GABA B receptor2 expression in the mouse cerebellar cortex.* Cerebellum, 2008. **7**(3): p. 295-303.
37. Ravanpay, A.C., S.J. Hansen, and J.M. Olson, *Transcriptional inhibition of REST by NeuroD2 during neuronal differentiation.* Mol Cell Neurosci, 2010. **44**(2): p. 178-89.
38. Fenster, S.D., et al., *Piccolo, a presynaptic zinc finger protein structurally related to bassoon.* Neuron, 2000. **25**(1): p. 203-14.
39. Bjorkblom, B., et al., *Constitutively active cytoplasmic c-Jun N-terminal kinase 1 is a dominant regulator of dendritic architecture: role of microtubule-associated protein 2 as an effector.* J Neurosci, 2005. **25**(27): p. 6350-61.
40. Cotter, L., et al., *Dlg1-PTEN interaction regulates myelin thickness to prevent damaging peripheral nerve overmyelination.* Science, 2010. **328**(5984): p. 1415-8.
41. Jin, K., et al., *Early B-cell factors are required for specifying multiple retinal cell types and subtypes from postmitotic precursors.* J Neurosci, 2010. **30**(36): p. 11902-16.
42. Oikawa, Y., et al., *Down-regulation of CIBZ, a novel substrate of caspase-3, induces apoptosis.* J Biol Chem, 2008. **283**(21): p. 14242-7.
43. Ohtsuka, T., et al., *Gene expression profiling of neural stem cells and identification of regulators of neural differentiation during cortical development.* Stem Cells, 2011. **29**(11): p. 1817-28.
44. Wang, Z.Z., et al., *Chemokine-like factor 1, a novel cytokine, induces nerve cell migration through the non-extracellular Ca2+-dependent tyrosine kinases pathway.* Brain Res, 2010. **1308**: p. 24-34.
45. Zheng, N., et al., *Structural basis of DNA recognition by the heterodimeric cell cycle transcription factor E2F-DP.* Genes Dev, 1999. **13**(6): p. 666-74.
